# The Chinese version of the Perceived Stress Questionnaire: development and validation amongst medical students and workers

**DOI:** 10.1186/s12955-020-01307-1

**Published:** 2020-03-13

**Authors:** Runtang Meng, Jingjing Li, Zhenkun Wang, Di Zhang, Bing Liu, Yi Luo, Ying Hu, Chuanhua Yu

**Affiliations:** 1 Department of Preventive Medicine, School of Health Sciences, Wuhan University, 185 Donghu Road, Wuhan, Hubei 430071 People’s Republic of China; 2 Department of Behavioral Sciences and Health Education, Rollins School of Public Health, Emory University, 1518 Clifton Road NE, Atlanta, GA 30322 USA; 3 Party Committee Organization Department, Tongji Hospital, Tongji Medical College, Huazhong University of Science and Technology, 1095 Jie Fang Avenue, Wuhan, Hubei 430030 People’s Republic of China; 4 Quality Control Department, Wuhan Asia General Hospital, 300 Taizi Lake North Road, Wuhan, Hubei 430056 People’s Republic of China; 5 Center of Health Administration and Development Studies, Hubei University of Medicine, 30 South Renmin Road, Shiyan, Hubei 442000 People’s Republic of China; 6 School of Nursing, Ningbo College of Health Sciences, 51 Xuefu Road, Ningbo, Zhejiang, 315100 People’s Republic of China; 7 Global Health Institute, Wuhan University, 8 South Donghu Road, Wuhan, Hubei 430072 People’s Republic of China

**Keywords:** Perceived stress, Instrument validation, Rasch analysis, Factor analysis

## Abstract

**Background:**

A valid and efficient stress measure is important for clinical and community settings. The objectives of this study were to translate the English version of the Perceived Stress Questionnaire (PSQ) into Chinese and to assess the psychometric properties of the Chinese version of the PSQ (C-PSQ). The C-PSQ evaluates subjective experiences of stress instead of a specific and objective status.

**Methods:**

Forward translations and back translations were used to translate the PSQ into Chinese. We used the C-PSQ to survey 2798 medical students and workers at three study sites in China from 2015 to 2017. Applying Rasch analysis (RA) and factor analysis (FA), we examined the measurement properties of the C-PSQ. Data were analyzed using the Rasch model for item fit, local dependence (LD), differential item functioning (DIF), unidimensionality, separation and reliability, response forms and person-item map. We first optimized the item selection in the Chinese version to maximize its psychometric quality. Second, we used cross-validation, by exploratory factor analysis (EFA) and confirmatory factor analysis (CFA), to determine the best fitting model in comparison to the different variants. Measurement invariance (MI) was tested using multi-group CFA across subgroups (medical students vs. medical workers). We evaluated validity of the C-PSQ using the criterion instruments, such as the Chinese version of the Perceived Stress Scale (PSS-10), the Short Form-8 Health Survey (SF-8) and the Goldberg Anxiety and Depression Scale (GADS). Reliability was assessed using internal consistency (Cronbach’s alpha, Guttman’s lambda-2, and McDonald’s omegas) and reproducibility (test–retest correlation and intraclass correlation coefficient, [ICC]).

**Results:**

Infit and/or outfit values indicated that all items fitted the Rasch model. Three item pairs presented local dependency (residual correlations > 0.30). Ten items showed DIF. Dimensionality instruction suggested that eight items should be deleted. One item showed low discrimination. Thirteen items from the original PSQ were retained in the C-PSQ adaptation (i.e. C-PSQ-13). We tested and verified four feasible models to perform EFA. Built on the EFA models, the optimal CFA model included two first-order factors (i.e. constraint and imbalance) and a second-order factor (i.e., perceived stress). The first-order model had acceptable goodness of fit (Normed Chi-square = 8.489, TLI = 0.957, CFI = 0.965, WRMR = 1.637, RMSEA [90% CI] = 0.078 [0.072, 0.084]). The second-order model showed identical model fit. Person separation index (PSI) and person reliability (PR) were 2.42 and 0.85, respectively. Response forms were adequate, item difficulty matched respondents’ ability levels, and unidimensionality was found in the two factors. Multi-group CFA showed validity of the optimal model. Concurrent validity of the C-PSQ-13 was 0.777, − 0.595 and 0.584 (Spearman correlation, *P* < 0.001, the same hereinafter) for the Chinese version of the PSS-10, SF-8, and GADS. For reliability analyses, internal consistency of the C-PSQ-13 was 0.878 (Cronbach’s alpha), 0.880 (Guttman’s lambda-2), and 0.880 (McDonald’s omegas); test–retest correlation and ICC were 0.782 and 0.805 in a 2-day interval, respectively.

**Conclusion:**

The C-PSQ-13 shows good metric characteristics for most indicators, which could contribute to stress research given its validity and economy. This study also contributes to the evidence based regarding between-group factorial structure analysis.

## Introduction

Stress has been as an old and a pivotal concept, but no commonly accepted definition of the term, in the health research since it is associated with various health outcomes and quality of life. Three prevailing approaches have been used by researchers to assess different aspects of this construct. Previous study concerning Selye’s response-based stress model assuming that events themselves act as the causal agent behind pathology, illness, cognitive impairment, maladaptive behavior, and other unhealthy outcomes; this model focuses on the assessment of the activation of specific physiological systems that are involved in the stress response [1, 2]. The stimulus model of stress, by comparison, emphasizes on the measurement of stressors in terms of environmental conditions (i.e. environmental stressors or stimuli) [3]. The transactional model of stress concentrates on the evaluation of the degree and type of the challenge, threat, harm, or loss, as well as on the individual’s perceived abilities to cope with such stressors [4]; the view to support this model implies, further, that stress is not the product of an imbalance between objective demands and response capacity, but rather of the perception of these factors [5, 6]. Although recognition around this general conceptualization over time, from which the construct of “perceived stress” arisen [7], the critical constructs underlying perceived stress have been more complex and challenging to evaluate.

As regards the measurement of stress, there is no clear consensus as to what the criteria should be for referral to measuring stressors in the case of objective conditions, including, but not limited to: (a) major life events and daily hassles (cumulative minor stressors) [3, 8, 9], (b) stress appraisal (perceptional processing) and/or emotional response [7, 10], (c) the coping and perceptions of control [11]. Indeed, the coping can be seen as a process, a strategy, and a response to all the elements (e.g., environment, individual disposition) that play a role in the effort to adapt [12]. No matter what kind of evaluation system, there are obvious drawbacks that limit their usefulness in past research.

Summers up the results of empirical research, accumulated or chronic stress has an adverse impact on mental well-being and physical health, whereas an important concern is that acute and temporally life events could not predict illness to the same extent [13], and what’s more, life events do not predict symptoms [14]. In addition, the personal impact of life events cannot be ascertained before the event actually occurred [15]. Recent stress research suggests that minor, chronic, daily stressors may be more important in determining outcomes than major life events [16]. Other approaches to measuring stress have diverted the focus from specific objective stressors to even more chronic and stress experiences independent of concrete objective occasion, known as a “subjectively experienced stress” [17]. Admittedly, inclination towards assessment of stress appraisal rather than stressful life event itself has since been targeted; more emphasis has been given to the development of stress measurement instruments that focused mainly on the subjective perception of the individual [7, 17–20].

Perceived stress is the feelings or thoughts that an individual has about how much stress they are experiencing at a given time or over a time period span, which reflects the interaction between an individual and environment [21]. Under such background, as an alternative instrument for assessing the perception of stress, studies increasingly have used the Perceived Stress Questionnaire (PSQ) of developers Levenstein and coworkers [22]. To understand the dimensionality of perceived stress, it has been aimed at overcoming some of the difficulties concerning the definition and selecting items tapping potential cognitive, emotional, and symptomatic sequelae of stressful events and circumstances, which tend to trigger or exacerbate disease symptoms [2, 23, 24]. The PSQ is specifically recommended for clinical settings, especially in psychosomatic medicine, though it has been employed in research studies as well. Similarly, another measuring stress perception tool, the Perceived Stress Scale (PSS) of developers Cohen and colleagues [7, 18], belongs to the most common instrument of this field in the literature. The original instrument (English) includes 14 items, and other forms have been evolved for 10- and 4- item subsets of the PSS over time; and it is currently translated into over 30 languages in accordance with Laboratory for the Study of Stress, Immunity, and Disease (Retrieved from: https://www.cmu.edu/dietrich/psychology/stress-immunity-disease-lab/index.html). The PSS items assess the extent to which respondents find their lives has been unpredictable, uncontrollable, and overloaded during the previous month. Moreover cannot but raise, what differs from the PSQ to the PSS is the specific nature of dimensions and elements, the former viewed affect and psychosomatic conditions as triggers of subsequent symptomatology and reflective of perceived stress, rather than as symptoms themselves, whereas the latter concerned about cognitive appraisal of stress and the respondent’s perceived control and coping capability [2, 18, 22, 23]. Again, both the PSQ and the PSS have been found to predict many psycho-physiological (psychological or physiological) outcomes that one would expect to follow from stress [25–34]. Accumulating research, expectantly, will continue and accelerate to focus on perceived stress in relation to health and disease over the upcoming years.

Other than the source language (English and Italian), there are multiple language versions of the PSQ currently, namely Swedish [35–37], Greek [38], German [23, 39], Spanish [40], Thai [41], Norwegian [25], French [42], Arabic [43] and Chinese [44]. Review of the literature suggests that in various cultures and countries, some of them provide relatively complete the psychometric properties, and others brief and incomplete, whereas the latter greater emphasis on clinical application. This tool contains two alternative forms, the General PSQ and the Recent PSQ, based upon respondent’s feelings and thoughts in a given time range, during the last two years or during the last month, respectively. The original PSQ has 30 items that distribute seven dimensions: harassment, overload, irritability, lack of joy, fatigue, worries and tension [22]. The Chinese version of the PSQ (C-PSQ) was tested only in nursing students in China, apart from some indicators of psychometric still existed with insufficiency [44]. Furthermore, longer questionnaires result in higher data collection costs and greater respondent burden and may lead to lower response rates and diminished quality of response [45]. Recent findings have suggested that the original PSQ in routine use could lead to respondent burden and has item redundancy [23, 37]. Specifically, the C-PSQ-30 likewise also needs to be parsimonious in order to keep the length of this scale as short as possible. As such, following previous research, this study examines two or more samples to evaluate the psychometric properties using Rasch analysis, factor analysis and other statistics methods through a psychologically comprehensive measurement.

## Method

### Measures

#### Perceived stress questionnaire (PSQ)

The PSQ was translated into Chinese using forward translations and back translations based on an integrated method and these guidelines [46–48], as described below:

Stage 1: Initial translation; two bilingual translators independently translated the original PSQ (English) into simplified Chinese.

Stage 2: Reconcile and synthesis of the translations; the researchers invite two translators and community experts (bicultural and bilingual individuals) to reconcile and synthesize the translations.

Stage 3: Back translation; using the synthetic version of the instrument from stage 2, another two bilingual translators separately translated it into English.

Stage 4: Expert committee; the ten-member expert panel and the original developer of the PSQ did review all the translations, reach a consensus on any discrepancy, and develop the pre-final version.

Stage 5: Pre-testing; during the internship, nine nursing students at the hospital participated pretest. Each student kindly completed the questionnaire (pre-final version). We, too, closely interviewed these participants to guarantee that there were no unintelligible or ambiguous questions. Finally, the final Chinese version of the Perceived Stress Questionnaire (C-PSQ) has been finalized.

Additionally, we emailed the final version to consult with Dr. Susan Levenstein to ensure that the two versions were equivalent in four levels: semantic, idiomatic, experiential and conceptual [48].

The C-PSQ is consistent with the original version of the PSQ (English) both in item order and scoring method, rating each item with reference to frequency of occurrence on a four-point Likert scale (1: almost never, 2: sometimes, 3: often, and 4: usually). Eight items (1, 7, 10, 13, 17, 21, 25, 29) need to be reverse scored. The PSQ index is calculated as (raw score - 30)/90, i.e. (raw score - the lowest possible score)/ (the highest possible score - the lowest possible score), which ranges from 0 to 1, with higher values indicating greater level of perceived stress.

#### Perceived stress scale (PSS)

As the PSS is short and easy to complete, it can be used together with other measures [49], thereby being selected as the criterion. Meanwhile, among three forms (number of items) of the PSS, it is recommended that the PSS-10 be used to measure perceived stress, both in practice and research [34, 50]. Given that the Simplified Chinese version of the PSS-10 (C-PSS-10) gained Dr. Cohens’ recognition [51], this form of the PSS was chosen in this survey. The C-PSS-10 consists of 10 the original PSS items in which the participants are asked to respond to each question on a five-point Likert scale (0 = never to 4 = very often), indicating how often they have felt or thought a certain way over the past 4 weeks. Six items (1, 2, 3, 6, 9, 10) are negative and the remaining four (4, 5, 7, 8) are positive, the latter are reverse scoring items. Composite scores can range from 0 to 40, with higher scores representing greater perceived stress.

#### Short form − 8 health survey (SF-8)

The SF-8 Health Survey (SF-8), a concise and generic assessment tool, especially in large-scale observational studies, generates a health profile consisting of eight sub-scales: physical functioning (PF), role limitations due to physical health problems (RP), bodily pain (BP), general health perceptions (GH), vitality (VT), social functioning (SF), role limitations due to emotional problems (RE), and mental health (MH), which are used for computing two summary measure scores (physical component score PCS and mental component score MCS) [52]. The SF-8 is comprised of an eight-item subset of the Short Form-36 Health Survey (SF-36) and has been translated into Chinese following the standard International Quality of Life Assessment (IQOLA) protocol in a prior study [53], whose Chinese version is repeatedly confirmed feasible, reliable, and valid using a large, representative sample from China and is readily available [54, 55]. The health dimensions used in our study are evaluated for physical health and mental health, which is scored with the Medical Outcomes Study scoring system [52]. Total scores are calculated as the weighted sum of the scores for all items, fluctuated in the range 0–100, with higher scores denoting better health.

#### Goldberg anxiety and depression scale (GADS)

The Goldberg Anxiety and Depression Scale (GADS), individually referred to as Goldberg Anxiety Scale (GAS) and Goldberg Depression Scale (GDS), is an 18-item self-report symptom inventory [56]. The global score, which ranges from 0 to 18, is based on responses (“yes” or “no”, with one or zero point respectively), asking how respondents to report symptoms experienced over the past month. Each subscale can give a maximum total of 9, with higher scores suggesting greater levels of symptomatology. Generally, anxiety score ≥ 5 or depression ≥2 shall be deemed as a 50% risk of a clinically important disturbance [56]. The GADS was selected as a comparator scale in earlier studies, which have revealed good psychometric properties and proven that it could be reliably and acceptably used by health sectors not specialized in mental health [57–59]. Based on the translation approaches mentioned above, the Chinese version of the GADS has been published elsewhere and displayed highly correlation with the C-PSQ in nursing students [44]. The various languages of the GADS presented a simple, quick and accurate method of detecting depression and anxiety in the general population.

### Setting and participants

The total sample size consisted of 2798 from three cities of China, i.e. Wuhan, Ningbo, and Shiyan, respectively corresponding to three samples named A, B, and C (Table 1). The participants were recruited from universities (colleges) and hospitals, which is closely related to medical field. Sample A and B belonged to medical students, while sample C was medical workers. A convenience sample of 130 undergraduate or postgraduate students at one university of public health, nursing, clinical or other medical related in Wuhan City participated in the survey. A total of 122 students in this sample completed the second test at last. Sampling method of sample B was stratified random sampling strategy and stratified college students by their grades. Briefly, we aimed to randomly sample 50% of the students from each grade of nursing students to obtain large, representative samples. Flowchart of the sampling strategy of sample B is shown in elsewhere [60]. Overall, a total of 1519 students from one college in Ningbo City were randomly selected. Sample C adopted stratified sampling to ensure maximal consideration of sampling representation by means of controlling their proportion of departments and occupational classes. Three hospitals in Shiyan City were randomly selected and this sample finally amounted to 1223 valid questionnaires for analysis. All participants were given a small incentive: a bar of chocolate or a pen worthy of 5 RMB (around 0.8 US dollars) for each responder as compensation for their time. Response rate: 93.85% for sample A, 95.66% for sample B and 90.59% for sample C. An average duration of the assessment for each respondent is about 15 min, and the top of the first page is printed with instructions for the questionnaire fulfillment. Sample test using the instruments was organized as follows. Using the C-PSQ and C-PSS-10, sample A was tested two times at two days interval (test-retest method). Sample B was measured by the C-PSQ and the Chinese GADS. Sample C was investigated through the C-PSQ, the Chinese SF-8, and the Chinese GADS (Table 1).

**Table 1:** Basic Statistics on Sample and Socio-demographic Characteristics of Participants

Variable	Total sample	Sample A	Sample B	Sample C
Time range	Nov 2015 to Jan 2017	Dec 2016 to Jan 2017	Nov 2015 to Jan 2016	Dec 2015 to Jan 2016
Location	Three cities	Wuhan	Ningbo	Shiyan
Composition	Medic	Postgraduates, undergraduates	Junior college students	Medical workers
Sampling method	Two ways	Convenient sampling	Stratified random sampling	Stratified random sampling
Response rates	2798/2999 (93.30)	122/130 (93.85)	1453/1519 (95.66)	1223/1350 (90.59)
Gender				
Male	397 (14.19)	42 (34.43)	20 (1.38)	335 (27.39)
Female	2401 (85.81)	80 (65.57)	1433 (98.62)	888 (72.61)
Age, years	24.97 ± 7.53	23.47 ± 2.65	19.58 ± 1.09	31.51 ± 7.07
PSQ Index	0.429 ± 0.155	0.402 ± 0.133^a^	0.399 ± 0.138	0.466 ± 0.168
PSS	–	15.689 ± 4.863^a^	–	–
Negative feelings	–	9.734 ± 3.506^a^	–	–
Positive feelings	–	5.955 ± 2.051^a^	–	–
SF-8	–	–	–	65.255 ± 17.097
PCS	–	–	–	67.145 ± 17.745
MCS	–	–	–	63.364 ± 18.924
GADS	–	–	8.081 ± 4.349	10.850 ± 4.691
GAS	–	–	4.503 ± 2.442	5.935 ± 2.460
GDS	–	–	3.577 ± 2.343	4.915 ± 2.620

Note: The above table demonstrated N (%) or Mean ± SD, SD = standard deviation; ^a^, by averaging scores of test-retest (two-time measurements)

### Statistical analysis

#### Item response theory (IRT)

Given that the responses are ordinal, we used item response model for analysis of the Chinese version of the PSQ. IRT application requires two important assumptions [61]: (1) the construct being measured is in fact unidimensional and (2) the items display local independence. As Georg Rasch noted, Rasch measurement generally converts dichotomous and rating scale observations into linear measures. In contrast to classical test theory, Rasch analysis accounts for both the difficulty of tasks (item difficulty) and the abilities of subjects (person ability) by modeling the relationship between a latent trait (i.e. a respondent’s functional ability) and the items used to measure that trait.

To validate the Chinese PSQ, these key indicators could best be summed up as: (1) Information-weighted fit (Infit) and outlier-sensitive fit (Outfit) mean square (MNSQ) statistics. Reasonable item mean square ranges for Infit and Outfit between 0.6 and 1.4 were considered as an indicator of acceptable fit, since type of test was rating scale (survey) [62]. (2) Unidimensionality. In addition to item-fit statistics, unidimensionality of the measured trait was assessed further using principal component analysis (PCA) of the residuals. There were two criteria: the variance explained by the first component should be adequate (> 50%); the unexplained variance in the first contrast of the residuals should be less than 3.0 eigenvalue units, preferably < 2.0 eigenvalue units [63]. (3) Local dependence (LD). Local item independence requires that an item be independent of other items - can be tested by the residual correlation between the items, with a cutoff value less than 0.30 [63]. Furthermore, following the latest recommendations, evaluation of local response dependence should also take into consideration the residual correlation relative to the average residual correlation [64]. (4) Person separation index (PSI) and person reliability (PR). Person separation is used to classify people. The ability of the scale to distinguish different strata (or groups) among participants was assessed using PSI and PR. They are indicators of the fit statistics’ reliability. An acceptable level of person separation of 2.0 and reliability of 0.8 corresponded to the ability to differentiate among 3 strata; while person separation of 3.0 and reliability of 0.9 respectively represents an excellent level or reliability. (5) Differential item functioning (DIF). Differential item functioning refers to the situation where members from different groups (e.g. different populations, gender, socioeconomic level) on the same level of the latent trait (disease severity, quality of life) have a different probability of giving a certain response to a particular item [65]. DIF contrast was considered absent if it was less than 0.50 logits (between − 0.50 and 0.50 logit values) [63], minimal but probably inconsequential if it ranged between 0.50 and 1.0 logits, and notable if it was > 1.0 logits. (6) Category thresholds. Category threshold order, which is reflected by the category probability curves, is an important parameter for demonstrating the usage of response categories, and it is essential for the calculation of person and item calibrations. Disorder thresholds occur when respondents have difficulty discriminating between ordered response options. (7) Person-item map. The map presents person measures ranked by their ability level and item difficulties ranked by difficulty. It can provide a way to visualize how well the items target the ability of the respondents. Optimally, the difference between respondents and item measure should be approximately 0 logits. Generally, a mean difference between the person and item measure in magnitude of 1.0 logits indicates significant mistargeting. (8) Discrimination index. The index of indiscrimination was defined as the ability of an item on the basis of which the discrimination is made between superiors and inferiors. Ebel and Frisbie gave following rule of thumb (i.e. 0.40 and up, very good items; 0.30 to 0.39, reasonably good but possibly subject to improvement; 0.20 to 0.29, marginal items, usually needing and being subject to improvement; below 0.19, poor items, to be rejected or improved by revision) [66] for determining the quality of items with respect to their discrimination index.

#### Classical test theory (CTT)

To evaluate the psychometric properties was an integral part of introducing a useful health measurement tool [67]. Validity was concerned with the true value and accuracy that a measure attempts to capture, and Reliability was defined as the consistency and precision of a measurement [68]. For validity evaluation work, we in turn assessed the construct validity, concurrent validity and convergent validity. The construct validity, factorial validation and the scale structure were verified through exploratory factor analysis (EFA) and confirmatory factor analysis (CFA) in aspects of exploration, validation and cross-validation. It would be better to split the sample and use one part of the data to derive a model and the other part to confirm the derived model. For exploratory analysis, Maximum Likelihood (ML) with an oblique rotation (promax, power coefficient = 4) were conducted, this choice of method for extraction and rotation was motivated by these prior studies [23, 37], and the number of components to retain was determined by eigenvalues (> 1), scree plots, items content and interpretability as well as total variance explained (usually 60% or higher) [69]. For confirmatory analysis, give that responses to items in the PSQ are obviously ordinal, we used a Weighted Least Square Mean and Variance Adjusted (WLSMV) to accommodate categorical data [70, 71]. Concurrent validity can be described as “scores on the measurement tool are correlated to a related criterion at the same time”; convergent validity can be defined as “extent to which different measures of the same construct correlate with one other” [72]. Concurrent validity and convergent validity were examined by testing Spearman’s correlations of the C-PSQ with the scales mentioned above. The correlative coefficient greater than or equal to 0.45 is recommended by many researchers [72]. We did not assess predictive validity and content validity. Content validity was reported elsewhere [44].

In CFA and/or multi-group CFA, some goodness-of-fit indices usually were recommend using benchmarks for judging model fit, such as Normed Chi-square (NC) < 2.0— < 3.0 [73], Non-Normed Fit Index/Tucker–Lewis Index (TLI) > 0.90 [69], Comparative Fit Index (CFI) > 0.90 [69], Root Mean Square Error of Approximation (RMSEA) < 0.05 (or 0.06 denotes “a good fit”) or 0.08 (denotes “a reasonable fit”) [74, 75], Weighted Root Mean Square Residual (WRMR) < 1.0 [76]. To compare the goodness-of-fit between the nested measurement invariance (MI) models, we followed the aforementioned recommendation of using differences in RMSEA, CFI, and TLI. Hereby, models with a change in CFI (ΔCFI) ≤ 0.010, change in RMSEA (ΔRMSEA) ≤ 0.015, and change in TLI (ΔTLI) ≤ 0.010 were favored [77–79]. Note that we did not compare with a chi-square difference test in four steps models, including configural equivalence, metric invariance, scalar invariance and strict invariance. Because the consensus was that this may be an overly stringent criterion since Δχ^2^ (χ^2^) test is dependent on sample size with a rejection of models with trivial practical misfit in large samples (*N* > 300) [78, 80, 81]. WRMR illustrated worse fit when sample size increased or model misspecification increased [76].

For reliability assessment, we first evaluated internal consistency using Cronbach’s alpha, Guttman’s lambda-2, McDonald’s omegas, item-total correlations, and split-half reliability coefficient. Cronbach’s alpha, Guttman’s lambda-2 (a better reliability estimation method [82]) and McDonald’s omegas (an optimum estimation on homogeneity reliability) are both internal reliability coefficients [83]. Item-total correlations offer information about how well each item is associated with total score for further assessment of internal consistency. Split-half reliability correlates scores between randomly divide all items that purport to measure the same construct into two sets, calculated based upon Spearman–Brown prediction formula in this study. Second, we evaluated the reproducibility, including test–retest reliability or score consistency over time using Pearson’s correlation and intraclass correlation coefficient (ICC) at an interval of two days. ICC estimates and their 95% confidence intervals were calculated selecting single measures and two-way mixed-effects model with absolute agreement type in view of method and range in collecting retest data [84, 85], to assess level of agreement between scores at two time points. We also computed the standard error of measurement, which helps quantify the variability of measurement errors and estimate measurement precision, as a supplement indicator in test–retest reliability assessment [86]. Cronbach’s alpha, a positive rating for internal consistency, reasonably ranges from 0.70 to 0.95 [87]. Considering the proof that alpha ≤ lambda-2 is a standard result in CTT [88, 89]; hence Guttman’s lambda-2 should move above 0.70. An omega value is above 0.70 indicates that there are a reliable total score [90]. Split-half reliability coefficient estimates above 0.70 are generally considered acceptable [91], obviously, it will be very close to 1.0. Item-total correlations should move in a range between 0.30 and 0.70 [92]. With respect to test–retest correlation and ICC, 0.70 or 0.75 would act as a set of recommended threshold values [72, 84, 87, 93].

The use of traditional methods, including CTT, was conducted using SPSS/PASW Statistics (version 18.0; SPSS Inc., Chicago, IL, USA), JASP (version 0.11.1; JASP Team, University of Amsterdam, Amsterdam, The Netherlands), and Mplus (version 7.4; Muthén & Muthén, Los Angeles, CA, USA). Among them, results in the confirmatory step were derived from Mplus based on polychoric correlation coefficients, other statistics were performed using SPSS and JASP. For item response theory analyses, the polytomous Rasch model based on joint maximum likelihood estimation (JMLE) was applied using Winsteps (version 4.4.6; John M. Linacre, Chicago, IL, USA).

### Ethics statement

Prior to launching this study, ethical approval was provided by the Ethics Committee of Wuhan University School of Medicine (WUSM), China. All procedures were in accordance with the relevant requirements of the Declaration of Helsinki and its revised version [94]. Informed consents were obtained from the relevant administrative department at the study site and from the medical students and workers enrolled. The data collection and transfer process were conducted anonymously to ensure full respect and protection of individual privacy rights. In addition, written permission to create and use this Chinese version of the PSQ was obtained from Susan Levenstein M.D. by e-mail.

## Results

### Participants of the study

The participants’ socio-demographic characteristics were shown in Table 1. As we can see, mean values and distribution of overall PSQ index in three samples were, in turn, 0.402 ± 0.133 (Sample A), 0.399 ± 0.138 (Sample B), 0.466 ± 0.168 (Sample C). Games-Howell tests (because of Levene Statistic F = 25.165, *P* < 0.001) revealed that the difference of between sample A and B was not statistically significant, Mean Difference (I-J) = 0.009, *P* = 0.781. More importantly, the differences were statistically significant in existing in between sample C and A, Mean Difference (I-J) = 0.059, *P* < 0.001; as well as sample C and B, Mean Difference (I-J) = 0.068, *P* < 0.001. Mean values and distribution of male and female were 0.468 ± 0.166 and 0.422 ± 0.153, t = 5.422, *P* < 0.001.

### Rasch analysis (item selection)

**Fig. 1:**
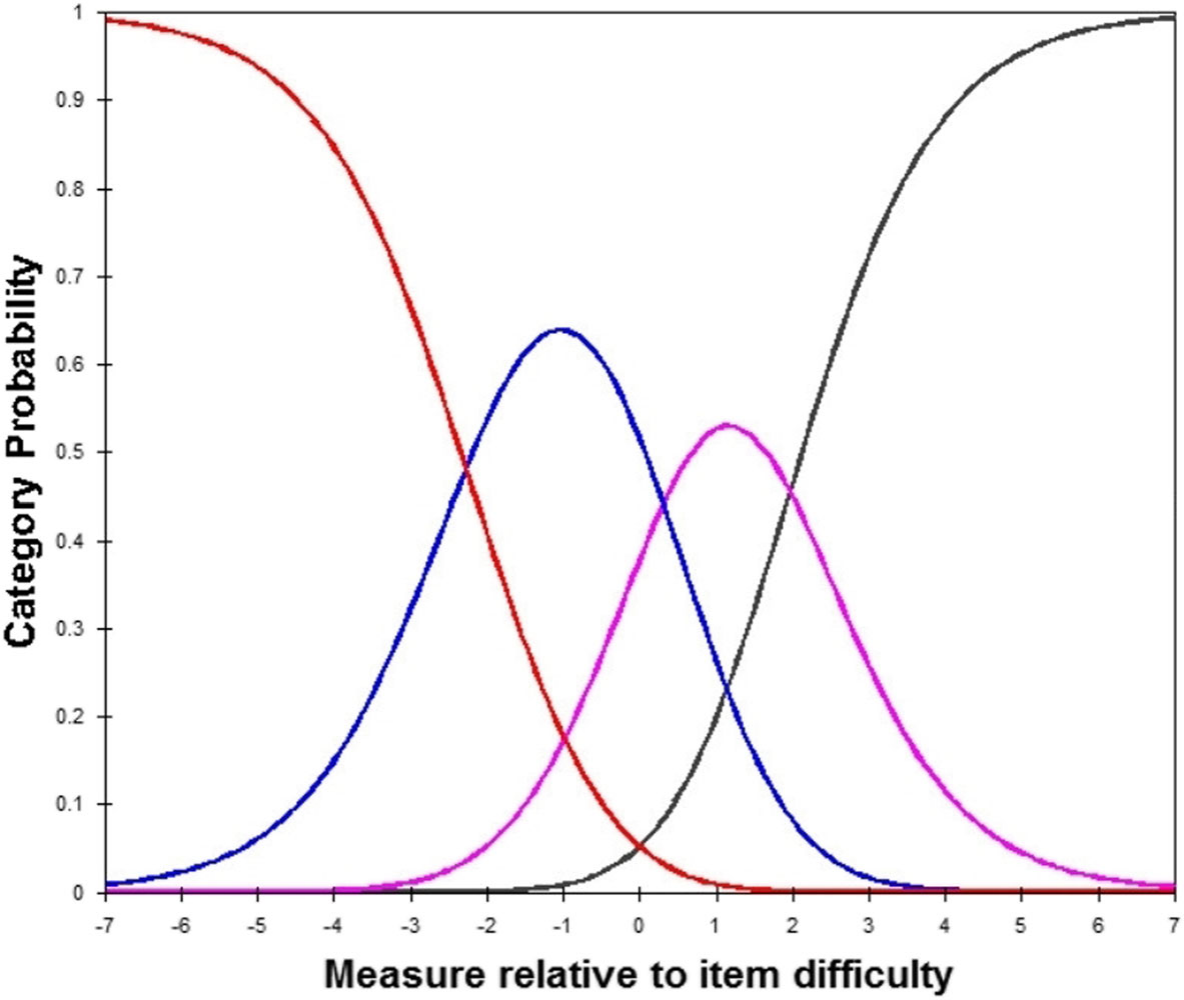
Category probability curves for the Chinese PSQ-30. This figure displays the category probability curves for the questionnaire which includes item 1 to 30, demonstrating ordered thresholds. The four curves from left to right represent 4 response categories (1 = almost never, 2 = sometimes, 3 = often, and 4 = usually)

**Fig. 2:**
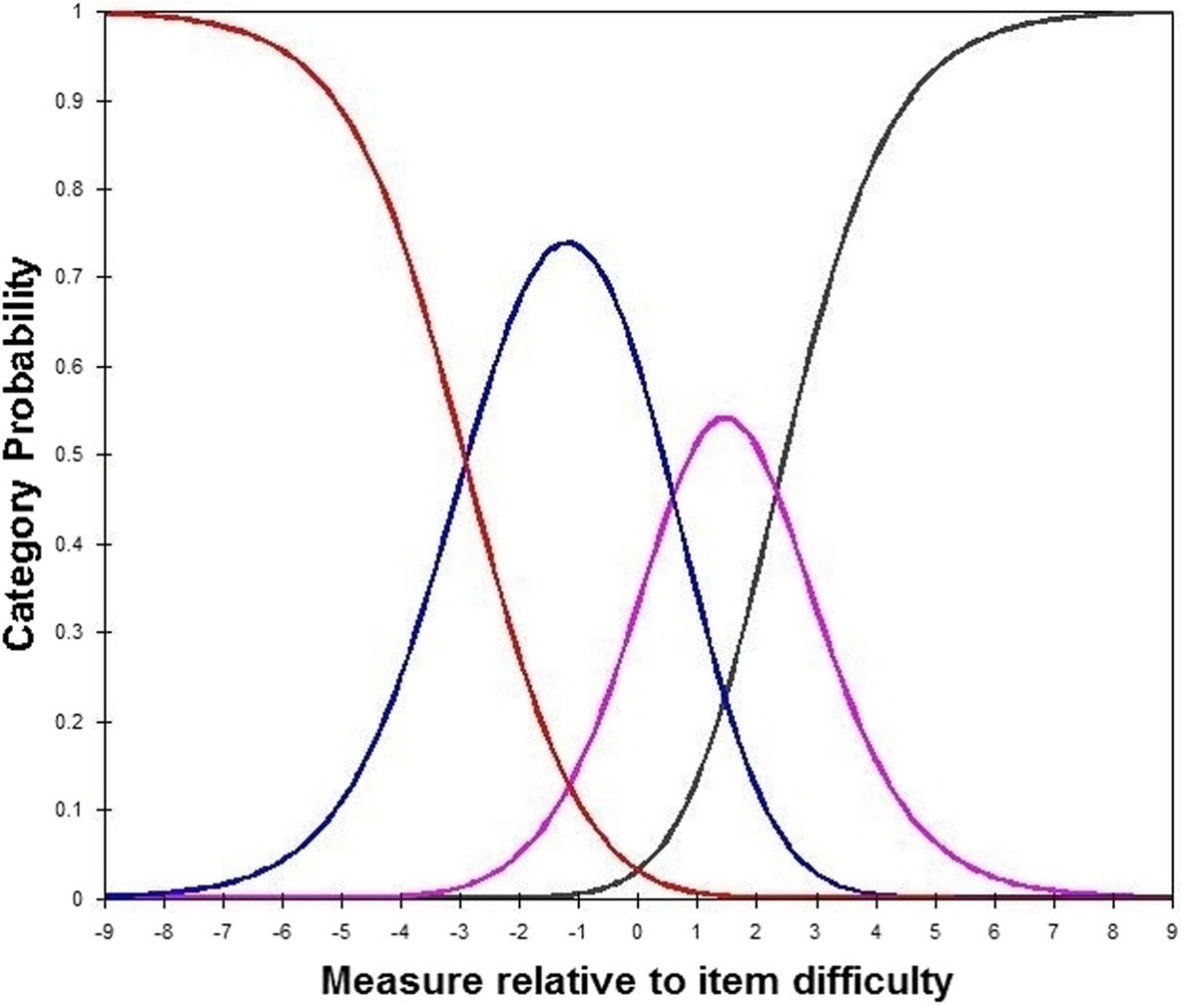
Category probability curves for the Chinese PSQ-13. This figure displays the category probability curves for the questionnaire which includes 13 items, demonstrating ordered thresholds. The four curves from left to right represent 4 response categories (1 = almost never, 2 = sometimes, 3 = often, and 4 = usually)

**Fig. 3:**
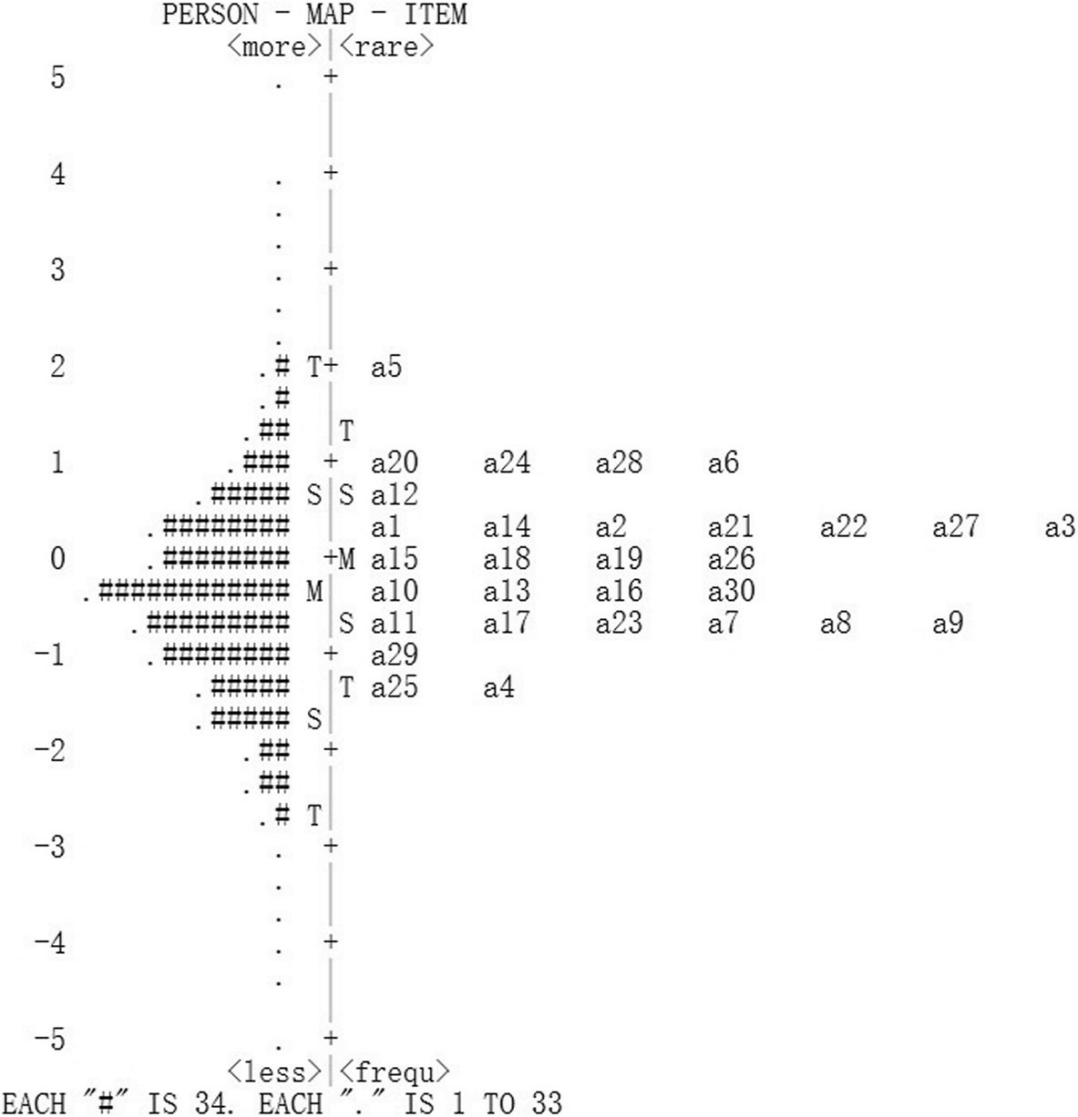
Person-item map of the Chinese PSQ-30

**Fig. 4:**
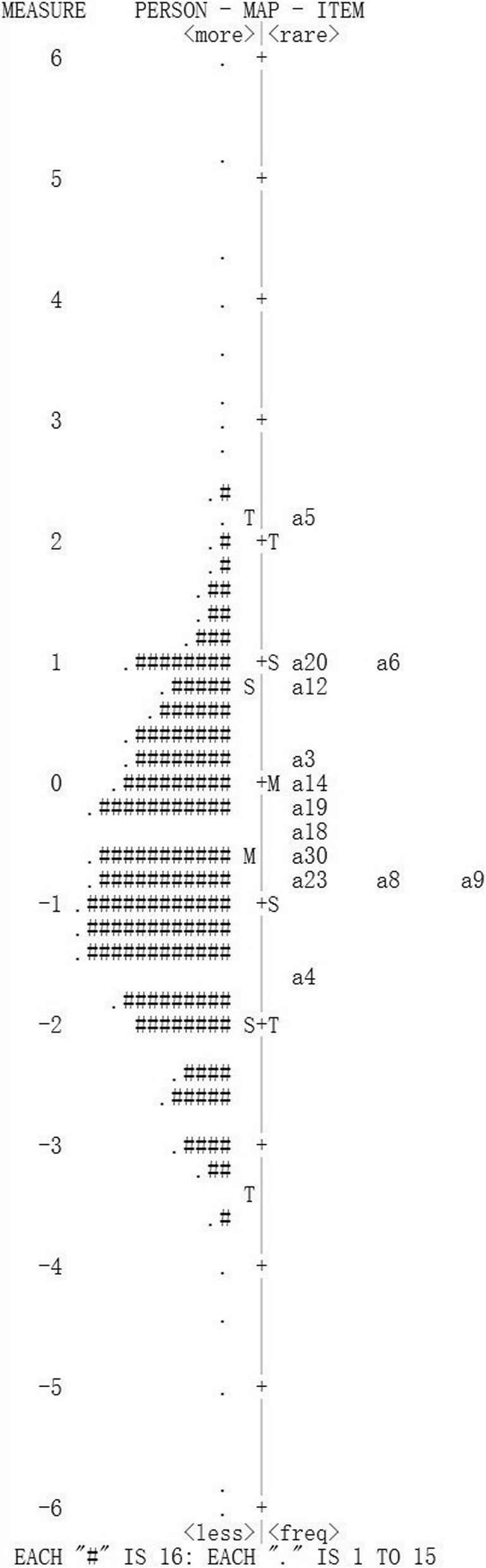
Person-item map of the Chinese PSQ-13

***Item fit statistics:*** Item fit statistics showed that almost all items fitted the Rasch model. No items were either under fitting (MNSQ > 1.4) or over fitting (MNSQ < 0.60) (Additional file 1, including Table 1a, b, c, d). ***Local dependence (LD)***: Three item pairs presented local dependency, i.e., displaying positive correlations of their residuals > 0.30. Compared to the average item residual correlation of − 0.033 in the thirty-item data set, the correlations between items one and thirteen of 0.312, items thirteen and twenty-one of 0.349, items twenty-six and twenty-seven were relatively large and these three item pairs were the positive correlation. ***Differential item functioning (DIF):*** In general, the items did not show DIF apart from (Additional file 2, including Table 2a, b, c, d): items 2, 7, 10, 22, 26, 27 (first round); items 15, 24, 28 (second round); items 16 (third round). ***Unidimensionality:*** The variance explained by RA ranged from 57.5 to 50.3% and unexplained variance in 1st contrast ranged from 3.21 to 1.70 (Table 2). In the first round, the instruction to delete these items is: 1, 7, 10, 13, 17, 21, 25, 29. ***Discrimination index***: Item 11 did show low discrimination index (0.37, below 0.40) in Table 1a. Finally, a total of seventeen items (i.e., item 1, 2, 7, 10, 11, 13, 15, 16, 17, 21, 22, 24, 25, 26, 27, 28, 29) should be removed. Then, the C-PSQ-13 was formed gradually by these above criterias. ***Separation and Reliability:*** Acceptable PSI (> 2.00) and good PR (> 0.80) values were respectively presented in Table 2, suggesting adequate separation ability for this instrument. ***Response forms:*** No evidence of disordered thresholds was found in the category probability curves for the C-PSQ-30 and C-PSQ-13, as the category calibration increased in an orderly way (demonstrated in Figs. 1 and 2), and suggesting this rating scale functioned well for both forms. Four response categories were found for all items, indicating three thresholds for each item. ***Person-item map:*** The person-item map given in Figs. 3 and 4 illustrated the relationship between item difficulty and person ability. In the C-PSQ-30 and C-PSQ-13, item difficulty had the same mean value = 0 logits, while person ability correspondingly had a mean value = − 0.43 logits and − 0.60 logits. Thus, the difference between the item and the person means were 0.43 logits and 0.60 logits respectively; both are less than 1.0 logit indicates targeting.

**Table 2:** Rasch Analysis among Different Items for the C-PSQ

	DIF	Discrimination	Dimensionality	Unexplained variance in 1st contrast	Total raw unexplained variance (%)	PSI	PR
PSQ-30	**2, 7, 10, 22, 26, 27**	**11**	**1, 7, 10, 13, 17, 21, 25, 29**	3.2109	57.5	3.45	0.92
PSQ-17	**15, 24, 28**	NR	Not	1.8736	50.9	2.85	0.89
PSQ-14	**16**	NR	Not	1.7727	50.9	2.52	0.86
PSQ-13	NR	NR	Not	1.7043	50.3	2.42	0.85
Cut-off	< 0.5	> 0.4	Based on 1st contrast	< 2 or < 3	> 50	> 2.0	> 0.8

Abbreviation: *DIF* differential item functioning, *PSI* person separation index, *PR* person reliability, *NR* not required

If item dropped (in bold) in DIF, Discrimination, Dimensionality;

PSQ-30 retained all 30 items;

PSQ-17 removed item 1, 2, 7, 10, 11, 13, 17, 21, 22, 25, 26, 27, 29;

PSQ-14 removed item 1, 2, 7, 10, 11, 13, 15, 17, 21, 22, 24, 25, 26, 27, 28, 29;

PSQ-13 removed item 1, 2, 7, 10, 11, 13, 15, 16, 17, 21, 22, 24, 25, 26, 27, 28, 29

### Factor analysis (construct validity)

Given sample size in factor analysis, at least 200 cases is probably an appropriate threshold, whereas samples of 500 or more observations are strongly recommended [95, 96]. Sampling adequacy for factor analysis was tested separately for medical students (sample A and B) and medical workers (sample C). In the C-PSQ-30, Kaiser–Meyer–Olkin (KMO) values were 0.951 (medical students) and 0.964 (medical workers); similarly, in the C-PSQ-13, KMO values were 0.923 (medical students) and 0.930 (medical workers), revealing marvelous level of sampling adequacy which were well above the recommended threshold of 0.6 [97, 98]. All of Bartlett’s test of sphericity were significant (*P* < 0.001), also denoting that the items could be considered apt for factor analyses. Inspection of eigenvalues, scree plot and item content and interpretability suggested respectively four-factor solution (30 items, medical students), five-factor solution (30 items, medical workers), two-factor solution (13 items, medical students) and two-factor solution (13 items, medical workers). The cross-validation was tested by the other sample set for the model.

In particular, the EFA of medical workers indicated that there is only one item (i.e. item 23) on a factor. The CFA model of medical students could not fit, thereby switching to principal component analysis in EFA. Table 3 compared the four models that showed the fit statistics. Through cross-validation, our CFAs found that the two-factor solution using medical students’ data to derive a model and using medical workers’ data to validate the derived model is the best fitting model. This optimal model has two factors, namely factor I (item 4, 8, 9, 14, 18, 19, 23, 30) and factor II (item 3, 5, 6, 12, 20). The two factors is inconsistency with that of the recently published literature [44], we renamed these factors “constraint” and “imbalance” respectively. The results of model fit are the same between in second-order model and first-order model owing to its two factors condition. Thereafter, we ran a series of CFA to test various factor structures reported in the literature, including English/ Italian (source language) [22], Spanish [40], German [23], Greek [38] and Swedish [37]. There existed relatively clear and distinct factor solution in these various versions and these were compared with our two-factor solution, Chinese. Table 3 presented the fit indices for all models tested.

**Table 3:** CFA of factorial structure solution among different conditions for the PSQ

	Factors	Items	CMIN	DF	*P*	NC	TLI	CFI	WRMR	RMSEA [90% CI]
Subgroups										
Medical Workers	4	30	2665.430	399	< 0.001	6.680	0.934	0.940	1.913	0.068 [0.066, 0.071]
Medicine Students^a^	5	30	3476.995	395	< 0.001	8.803	0.903	0.912	2.324	0.070 [0.068, 0.073]
Medical Workers^*^	**2**	**13**	**543.294**	**64**	**< 0.001**	**8.489**	**0.957**	**0.965**	**1.637**	**0.078 [0.072, 0.084]**
Medicine Students	2	13	607.965	64	< 0.001	9.499	0.950	0.959	1.792	0.073 [0.068, 0.079]
Languages										
Chinese	1	13	1443.451	65	< 0.001	22.207	0.940	0.950	2.687	0.087 [0.083, 0.091]
**Chinese**	**2**	**13**	**936.631**	**64**	**< 0.001**	**14.635**	**0.961**	**0.968**	**2.148**	**0.070 [0.066, 0.074]**
English	7	30	11,042.264	384	< 0.001	28.756	0.836	0.855	4.027	0.100 [0.098, 0.101]
Spanish	6	30	9471.091	390	< 0.001	24.285	0.862	0.877	3.637	0.091 [0.090, 0.093]
German	4	20	5069.364	164	< 0.001	30.910	0.873	0.890	3.693	0.103 [0.101, 0.106]
Greek	5	30	8237.623	395	< 0.001	20.855	0.883	0.893	3.390	0.084 [0.083, 0.086]
Swedish	5	21	5737.159	179	< 0.001	32.051	0.855	0.877	3.776	0.105 [0.103, 0.108]
Cutoff value	N/A	N/A	N/A	N/A	> 0.05	< 2— < 3	> 0.90	> 0.90	< 1.0	< 0.05 or 0.08

Note: *CMIN* chi-square; *DF* degrees of freedom; *NC* normed chi-square, CMIN/DF; *TLI* Tucker-Lewis index; *CFI* comparative fit index; *WRMR* weighted root mean square residual; *RMSEA* root mean square error of approximation; *N/A* not applicable

*Best fitting model (in bold), ^a^ CFA in Medical Students was used to test a model derived using EFA in Medical Workers, extraction method: Principal Component Analysis; because of using the maximum likelihood method, there were 5 dimensions that can be obtained, one of which has only one item. Others extraction method: Maximum Likelihood

The CFA of different languages used total sample (three samples, Using the data of sample A for the first time has to be merged into the total sample.); the Swedish version (Rönnlund et al., 2015), the Greek version (Karatza et al., 2014) and the German version (Fliege et al., 2005), the Spanish version (Sanz-Carrillo et al., 2002), the English/original version (Levenstein et al., 1993)

Next, results regarding measurement invariance of the C-PSQ-13 across subgroups (medical students and medical workers) are presented in Table 4. The results of four steps ranging from least to most rigorous suggested invariance across subgroups: ΔTLI = 0.002, 0.009, and 0.000 < 0.01; ΔCFI = 0.004, 0.014, and 0.006; ΔRMSEA = 0.001, 0.004, and 0.000 < 0.015. In consideration of subgroups, the C-PSQ-13 can be considered fully invariant (except for 0.014, as described above). In view of sample size across gender and age is too unbalance, therefore we no performed Multi-group CFA in these between groups.

**Table 4:** Measurement Invariance of the C-PSQ across Subgroups

Two-factor	NC	TLI	CFI	RMSEA	Δχ^2^	ΔDF	ΔTLI	ΔCFI	ΔRMSEA
M1:Configural invariance	799.864/128	0.929	0.942	0.061 [0.057, 0.065]					
M2:Metric invariance	855.073/141	0.931	0.938	0.060 [0.056, 0.064]	55.209	13	0.002	0.004	−0.001
M3:Scalar invariance	1023.508/152	0.922	0.924	0.064 [0.060, 0.068]	168.435	11	−0.009	− 0.014	0.004
M4:Strict invariance	1109.198/165	0.922	0.918	0.064 [0.060, 0.068]	85.690	13	0.000	−0.006	0.000
Cutoff value	< 2— < 3	> 0.90	> 0.90	< 0.05 or < 0.08	N/A	N/A	≤0.010	≤0.005 or ≤ 0.010	≤ 0.015

Abbreviation: *NC* normed chi-square, CMIN/DF; *TLI* Tucker-Lewis index; *CFI* comparative fit index; *RMSEA* root mean square error of approximation; *DF* degrees of freedom; Δ a change in (χ^2^, DF, TLI, CFI, RMSEA); *N/A* not applicable

### Concurrent and convergent validity

The Chinese PSS-10, SF-8 and GADS would serve as a criterion separately. The correlation matrix of these instruments was depicted as follows (Table 5). The correlation coefficient between subscales (scale) of the C-PSQ-13 ranged from 0.640 to 0.947, indicating moderate (0.5–0.8) to high correlation. Most of correlation coefficients were above quality criteria (0.45) except for between GAS and imbalance (r = 0.438) in these instruments and its subscales. Especially concerning was that all subscales and the C-PSQ-13 most highly correlated with the Chinese PSS-10 in these criterions whereas the Chinese GADS reflected the lowest correlation with the C-PSQ-13 and its subscales. Coefficients of correlation negative feelings with the C-PSQ-13 and its subscales were higher than positive feelings with the C-PSQ-13 and its subscales, MCS and PCS with similar results. Additionally, the Chinese SF-8 and the C-PSQ-13 and its subscales were negatively correlated. The results demonstrated that scores of other instruments highly correlated with PSQ Index. On the whole, concurrent and convergent validity of the C-PSQ-13 and its subscales was more satisfactory.

**Table 5:** Concurrent Validity and Convergent Validity for the C-PSQ-13 and Its Subscales Intercorrelations

	PSQ-13	Constraint	Imbalance
PSQ-13^a^		0.947	0.843
Constraint			0.640
Imbalance			
PSS-10^b^	0.777	0.709	0.697
Positive feelings	0.533	0.479	0.476
Negative feelings	0.773	0.713	0.689
SF-8^c^	−0.595	−0.571	−0.510
PCS	−0.482	−0.466	−0.414
MCS	−0.619	−0.592	−0.534
GADS^d^	0.584	0.559	0.492
GAS	0.534	0.518	0.438
GDS	0.542	0.513	0.469

Note:

All Spearman correlations *P* < 0.001;

Recode reverse-coded items;

^a^, N = 2798, Sample A (first time), B and C; ^b^, *N* = 122, Sample A (by averaging scores of test-retest); ^c^, *N* = 1223, Sample C; ^d^, *N* = 2676, Sample B and C;

PSS’s Guttman’s lambda-2 (first time): Positive feelings = 0.710, Negative feelings = 0.773, whole scale = 0.800; PSS’s Guttman’s lambda-2 (second time): Positive feelings = 0.677, Negative feelings = 0.867, whole scale = 0.861; SF-8’s Guttman’s lambda-2: PCS = 0.815, MCS = 0.858, whole scale = 0.898; GADS’s Guttman’s lambda-2: GAS = 0.780, GDS = 0.789, whole scale = 0.870

### Reliability

Table 6 summarized the instrument distribution and the reliability test results based on quality criteria. Of these, adequate item-total score correlations ranges between 0.30 and 0.70, as described in CTT. All corrected item-total correlations were in range (except for item 11, r = 0.319), reflecting satisfactory scale homogeneity. Note that if item 11 dropped, Cronbach’s alpha and McDonald’s omegas on the PSQ would be increased. Cronbach’s alpha of the Chinese both PSQ-13 and PSQ-30 were 0.878 and 0.935 respectively. Both McDonald’s omegas and Guttman’s lambda-2 were the same result, 0.880 and 0.937 respectively. Split-half reliability coefficients were 0.852 and 0.919 individually. Additionally, internal consistency reliability of subscales, using Cronbach’s alpha, Guttman’s lambda-2 and McDonald’s omegas respectively, were 0.834, 0.835, 0.838 (constraint) and 0.762, 0.765, 0.764 (imbalance). These indicators indicated good internal consistency reliability on whole scale and its subscales. Still have, for reproducibility over time, the Spearman’s correlation between time points and the ICCs for absolute agreement were 0.782 vs. 0.874, 0.805 vs. 0.899. Overall, the test–retest reliabilities of both scales met the quality criterion. The standard errors of measurement were 0.070 vs. 0.049 in the C-PSQ-13 and C-PSQ-30, as well as with lower precision accuracy in the former.

**Table 6:** Reliability of the Chinese between PSQ-13 and PSQ-30 (*N* = 2798)

	Quality criteria	PSQ-13	PSQ-30
Mean ± SD^a^	N/A	0.414 ± 0.158	0.429 ± 0.155
Item-total correlation	0.30–0.70	0.453–0.688	0.319–0.698
Cronbach’s alpha (α)	0.70–0.95	0.878	0.935
Guttman’s lambda-2 (λ_2_)	> 0.70	0.880	0.937
McDonald’s omegas (ω)	> 0.70	0.880	0.937
Split-half reliability coefficient	> 0.70	0.852	0.919
Test–retest correlation (*N* = 122)^b^	> 0.70	0.782 [0.679, 0.853]	0.874 [0.800, 0.920]
Intraclass correlation coefficient (ICC) for absolute agreement (N = 122)^b^	> 0.75 or 0.70	0.805 [0.729, 0.861]	0.899 [0.858, 0.929]
Standard error of measurement^c^	N/A	0.070	0.049

Note:

*N/A* not applicable;

^a^, The PSQ Index was used, SD = standard deviation; Sample A (N = 122, first time) has to be merged into total sample (N = 2798);

^b^, 95% Confidence Interval estimation of test-retest correlation used bootstrap, all Spearman correlation *P* < 0.001;

^c^, Standard error of measurement was calculated as SD × sqrt (1-ICC)

## Discussion

The PSQ was developed in 1993 to examine people subjective stress perception on different clinical or non- clinical areas, including both physical and psychological on quality of life. The results of a Rasch analysis and a factor analysis were complementary, which helped provide a comprehensive perspective on the construct validity of the Chinese PSQ. The previous study validated the measurement properties of the Chinese PSQ by CTT only [44]. Admittedly short instruments (scales or questionnaires) improve assessment as they save response time and effort, increase response rate, minimize burden, and decrease fatigue effect. The development and validation was performed using Rasch analysis, a relatively modern psychometric technique for developing and refining rating instruments (i.e. scales and questionnaires) with sound psychometric properties. Indeed, since both multidimensionality and response dependency are serious threats of the metric characteristics of an assessment and implies that responses to an item depend on responses to other items or that the scale reflects more than one latent trait, requiring support for unidimensionality and local independence [99, 100]. Thus, IRT methodology application is contingent on the extent to which these assumption are met [61]. The results (first round) of the Rasch model analysis revealed that the C-PSQ-30 is not unidimensional, since the unexplained variance in the first contrast (3.21) was greater than 2.0 in the PCA. Summary of previous study on the validation of the PSQ showed that this instrument may be subjectively conceived as a seven-factor model [22], six-factor model [40], five-factor model [37, 38], or four-factor model [23]. Our current study indicated that three pair items showed local dependency, six items (first round) presented DIF and one item demonstrated low discrimination index. According to the assumptions and guidelines [61, 63], we finally performed four round validation until that are met. Of these, we removed 10 items by three rounds of DIF. It would not be more reasonable to build an instrument that is not biased (with items that do not present a Differential Item Functioning). Crucially, 13 items were retained in the Chinese PSQ adaptation (Table 2). Rasch reliability indexes (PSI and PR) confirmed their high values, which give us a good degree of confidence in the consistency of both person-ability and item-difficulty estimates. Our study demonstrated an ordered threshold in the category probability curves, which means that the response forms were adequate, the item difficulty matched medical students’ or medical workers’ (these respondents’) ability levels. The items were well-targeted to the subjects, with a mean difference of 0.43 and 0.60 logits in C-PSQ-30 and C-PSQ-13, respectively. This means that the difficulty of the items on these questionnaires were appropriate for the ability of respondents.

The focus of the present study was to investigate a more appropriate factorial structure of the C-PSQ, especially to improve and promote this Chinese PSQ adaptation. The analyses encompassed the EFA to extract factors, the CFA to test model, and cross-validation of the model seen as suitable in separate large-scale samples. Regarding exploratory factor analysis among medical students, two factors were extracted from the C-PSQ-13. This model is the best fitting model. Pertaining to WRMR, the smaller value, the better fit (acceptable < 1, and good < 0.8 [101]), as Linda K. Muthén noted in 2005, in some cases other fitting indices were good, and the WRMR value is large, so we did not focus on WRMR at that time. Notwithstanding PSQ Index was originally proposed by the instrument developers and counted to a perceived stress index across the PSQ items [22], it is notable that the model established in this study is to continue supporting a perceived stress factor, that reflects all first-order factors [37, 38], and confirms that on utilization of PSQ Index do have a certain rationality and feasibility. According to the results of current study and previous studies, this Chinese version (C-PSQ-13), the Swedish version [37] and the German version [23] belonged to the reduced version, whereas the Greek version [38], the Spanish version [40], the Thai version [41], the Norwegian version [25] and the Arabic version [43] retained all 30 items, while its various versions still remained adaptation on levels of items and factors. Upon closer inspection, the structure of the questions in each subscale differed from those of the original instrument. Indeed, these across studies that evaluated the factor structures have reported non-unidimensional for the PSQ. Based on the Recent PSQ rather than the General PSQ form of the questionnaire could possibly have affected the outcome in our study. These conditions, cultural adaptation and translation quality as well as sample properties, would be unable to ignore for influence on factor solution. Cross-cultural differences, perhaps not surprisingly, led to discuss some discrepancy on factor structures of the PSQ.

Criterion validation consists of correlating the new instrument with well accepted measure of the same characteristics, usually known as the criterion validity. Using the Chinese PSS-10, SF-8 and GADS respectively as the criterion, concurrent validation values of the C-PSQ-13 are above a reasonable threshold value (0.45) [72]. More specifically, the correlation with the Chinese PSS is close to 0.80 (high correlation ≥0.80), which revealed some aspect of the new tool with a widely accepted measure of the same characteristics [67]. Predictive validity was failed to assess on account of no follow-up.

A satisfactory level of reliability depends on how a measure is being used. Three internal consistency reliability methods of this reduced version are less than that of the C-PSQ-30, but still display good reliability. Cronbach’s alpha values were higher than 0.70 for the C-PSQ-13 and the C-PSQ-30 in the present study, like across studies and then their alpha values held wave nearby 0.90 [22, 23, 25, 36, 38–41]. The higher alpha values in those studies may be owing to characteristics of the samples. The more items would too have higher Cronbach’s alpha values. Guttman’s lambda-2 values, only reported in this study, still were greater than quality control standard for the C-PSQ-13 and the C-PSQ-30. McDonald’s omegas values are approximately equal to Guttman’s lambda-2 values for the C-PSQ-13 and the C-PSQ-30, respectively. Alpha is and remains to be the best choice among all published reliability coefficients, even though alpha should be replaced by better and readily available methods [82, 102]. Hence, we decided to report both alpha and lambda-2, as an indication of internal consistency. Their samples of different studies, at any rate, appeared to have experienced relatively intense stress and thus may have responded to items more consistently. Although internal consistency can be higher in the present study, on most occasions, additional evaluations such as item-total correlations or split-half reliability coefficients were suggested to confirm the internal consistency of the C-PSQ-13.

With regard to reproducibility, the aim was to assess reliability and agreement, through repeated measurements in stable respondents (test–retest) provide similar answers. Notably, test–retest Spearman correlations of the adaptation and the Chinese PSQ are apparently greater than quality criteria. Relatively, these values (0.782 and 0.874) are more than the results at one-week intervals in the former research [44]. Test–retest Spearman correlation of the C-PSQ-13 is less than the original study at 8 days, the Spanish study at 13 days, the Greek study at one month, whereas the result of the Chinese PSQ is more than that of three studies [22, 38, 40]. These results proved that the instrument has an appropriate level of both stability and responsiveness to change over time. Although test–retest reliability are commonly measured with Spearman correlation, it is better to use the intraclass correlation based on a two-way repeated measures analysis of variance looking at absolute agreement, since this is sensitive to any bias between or among times [67]. ICCs of this adaption and the C-PSQ were above 0.75 and close to 0.90 respectively, indicating good and excellent reliability [84]. The score reproducibility over time of the adaption (*r*_*s*_ = 0.782, ICC = 0.805) was less than that of the C-PSQ (*r*_*s*_ = 0.874, ICC = 0.899) in 122 participants in this study. In brief, the relatively high internal consistency (alpha, lambda-2, omegas) and reproducibility (test–retest correlations, ICCs) values disclosed strong reliability.

In summary, the results of the present study validated the metric characteristics of the revised PSQ, the Simplification of the PSQ-13, which was adapted from the original PSQ-30. Through examination of a series of results, the C-PSQ-13 obtains good and stable psychometric properties for most indicators and still remains confirmed in current study. To date, no known studies have examined measurement properties of the PSQ using IRT, in combination with CTT. However, this study has several limitations. First, all voluntary samples originated from the medic field, possibly resulting in insufficient sample representativeness and the lack of external validity in our work. In other words, it could limit its generalizability. Second, the study focuses on the fit of Rasch model, item section, construct validity, internal consistency and test–retest reliability. Other forms validity (predictive, content) is needed to more fully support metric characteristics of the instrument. While the values of ICCs in the testing of test–retest reliability were greater than 0.75, securing reliability, the sample size of 122 (only sample A) respondents apparently was a little small. Third, we cannot exclude that some characteristics (such as cross-cultural and language differences, translation quality, sampling attributes, testing situations, forms of instrument [the Recent or General PSQ] and other subjective and objective factors [37, 103]) influenced our results. Lastly, most of the data were cross-sectional, thereby limiting the capability of drawing causal inferences. As such, further research should replicate these findings with other populations by adequate follow-up data and/or multi-center studies concerning stress perception.

## Conclusion

Taken together, the C-PSQ-13 attained to a valid, reliable, cost and time-effective measuring tool that enables us to evaluate perceived stress both in respect to research studies and clinical settings. It measures two dimensions including constraint and imbalance. The best model is to continue supporting a perceived stress factor and to validate measurement invariance across subgroups, confirming that on utilization of PSQ Index do have a certain rationality and feasibility.

Results contribute to the emerging empirical comparison across studies and/or subgroups concerning the factorial structure of the PSQ. Various studies can be compared with the reference values at hand, such as PSQ Index and different solutions on factor structure from the original. Admittedly, the various language versions of the PSQ, including the original PSQ’s structure, were not replicable. Nevertheless, our revision of the PSQ’s structure proved relative stability in Chinese language and culture. In consideration of this advantage and respondent burden, the C-PSQ-13 is preferable, as a potentially valuable instrument.

## Supplementary information

**Additional file 1 **Table 1a Rasch Analysis of Item Statistics for the C-PSQ-30 (*N* = 2798). Table 1b Rasch Analysis of Item Statistics for the C-PSQ-17 (N = 2798). Table 1c Rasch Analysis of Item Statistics for the C-PSQ-14 (N = 2798). Table 1d Rasch Analysis of Item Statistics for the C-PSQ-13 (N = 2798).

**Additional file 2.** Table 2a Differential Item Functioning of the C-PSQ-30 across Subgroups. Table 2b Differential Item Functioning of the C-PSQ-17 across Subgroups. Table 2c Differential Item Functioning of the C-PSQ-14 across Subgroups. Table 2d Differential Item Functioning of the C-PSQ-13 across Subgroups.

## Data Availability

Requests for the formatted C-PSQ and its scoring rubric (available at no charge for research purposes) should be directed to the first author at mengruntang@whu.edu.cn or mengruntang@163.com. Due to ethical restrictions, participant-level data cannot be made publicly available. The datasets performed during the current study are available from the first author on reasonable request.

## Abbreviations

AIC: Akaike information criterion
CFA: Confirmatory factor analysis
CFI: Comparative fit index
CTT: Classical test theory
DIF: Differential item functioning
EFA: Exploratory factor analysis
FA: Factor analysis
GADS: Goldberg Anxiety and Depression Scale
GAS: Goldberg Anxiety Scale
GDS: Goldberg Depression Scale
ICC: Intraclass correlation coefficient
IRT: Item response theory
JMLE: Joint maximum likelihood estimation
KMO: Kaiser–Meyer–Olkin
LD: Local dependence
MCS: mental component score
ML: Maximum likelihood
MNSQ: Mean square
NC: Normed chi-square, CMIN/DF
PCA: Principal component analysis
PCS: Physical component score
PR: Person reliability
PSI: Person separation index
PSQ: Perceived Stress Questionnaire
PSS: Perceived Stress Scale
RA: Rasch analysis
RMSEA: Root mean square error of approximation
SF-8: Short Form-8 Health Survey
SRMR: Standardized root mean residual
TLI: Tucker-Lewis index
WLSMV: Weighted least square mean and variance adjusted
WRMR: Weighted root mean square residual

## References

[1] Szabo S, Tache Y, Somogyi A (2012). The legacy of Hans Selye and the origins of stress research: a retrospective 75 years after his landmark brief “letter” to the editor# of nature. Stress.

[2] Lehman KA, Burns MN, Gagen EC, Mohr DC (2012). Development of the brief inventory of perceived stress. J Clin Psychol.

[3] Holmes TH, Rahe RH (1967). The social readjustment rating scale. J Psychosom Res.

[4] Lazarus RS, Folkman S (1984). Stress: appraisal and coping.

[5] Alvarenga ME, Byrne DG (2016). Handbook of psychocardiology. Stress concepts, models, and measures.

[6] Monroe SM (2008). Modern approaches to conceptualizing and measuring human life stress. Annu Rev Clin Psychol.

[7] Cohen S, Kessler RC, Gordon LU (1997). Measuring stress: a guide for health and social scientists.

[8] Dohrenwend BP, Shrout PE. "Hassles" in the conceptualization and measurement of life stress variables. 1985;40(7):780–5.

[9] Kanner AD, Coyne JC, Schaefer C, Lazarus RS (1981). Comparison of two modes of stress measurement: daily hassles and uplifts versus major life events. J Behav Med.

[10] Lazarus RS (2006). Stress and emotion: a new synthesis.

[11] Fleming R, Baum A, Singer JE (1984). Toward an integrative approach to the study of stress. J Pers Soc Psychol.

[12] Krabbe P (2016). The measurement of health and health status: concepts, methods and applications from a multidisciplinary perspective.

[13] Searle A, Bennett P (2001). Psychological factors and inflammatory bowel disease: a review of a decade of literature. Psychol Health Med.

[14] Grant I, Patterson T, Olshen R, Yager J (1987). Life events do not predict symptoms: symptoms predict symptoms. J Behav Med.

[15] Dohrenwend BS, Dohrenwend BP (1981). Socioenvironmental factors, stress, and psychopathology. Am J Community Psychol.

[16] Fink G (2007). Encyclopedia of stress.

[17] DeLongis A, Coyne JC, Dakof G, Folkman S, Lazarus RS (1982). Relationship of daily hassles, uplifts, and major life events to health status. Health Psychol.

[18] Cohen S, Kamarck T, Mermelstein R. A global measure of perceived stress. J Health Soc Behav. 1983:385–96. 6668417

[19] O'Keeffe MK, Baum A (1990). Conceptual and methodological issues in the study of chronic stress. Stress Med.

[20] Cohen S. Contrasting the Hassles Scale and the Perceived Stress Scale: Who's really measuring appraised stress? 1986;41(6):716–8.

[21] Phillips AC, Gellman MD, Turner JR (2013). Perceived stress. Encyclopedia of behavioral medicine.

[22] Levenstein S, Prantera C, Varvo V, Scribano ML, Berto E, Luzi C (1993). Development of the perceived stress questionnaire: a new tool for psychosomatic research. J Psychosom Res.

[23] Fliege H, Rose M, Arck P, Walter OB, Kocalevent R-D, Weber C (2005). The perceived stress questionnaire (PSQ) reconsidered: validation and reference values from different clinical and healthy adult samples. Psychosom Med.

[24] Shahid A, Wilkinson K, Marcu S, Shapiro C (2012). STOP, THAT and one hundred other sleep scales.

[25] Østerås B, Sigmundsson H, Haga M (2015). Perceived stress and musculoskeletal pain are prevalent and significantly associated in adolescents: an epidemiological cross-sectional study. BMC Public Health.

[26] Zunhammer M, Eichhammer P, Busch V (2014). Sleep quality during exam stress: the role of alcohol, caffeine and nicotine. PLoS One.

[27] Crowe S, Barot J, Caldow S, d’Aspromonte J, Dell’Orso J, Di Clemente A (2011). The effect of caffeine and stress on auditory hallucinations in a non-clinical sample. Personal Individ Differ.

[28] Öhman L, Bergdahl J, Nyberg L, Nilsson LG (2007). Longitudinal analysis of the relation between moderate long-term stress and health. Stress Health.

[29] Levenstein S, Prantera C, Varvo V, Scribano ML, Andreoli A, Luzi C (2000). Stress and exacerbation in ulcerative colitis: a prospective study of patients enrolled in remission. Am J Gastroenterol.

[30] Levenstein S, Prantera C, Varvo V, Scribano ML, Berto E, Andreoli A (1994). Psychological stress and disease activity in ulcerative colitis: a multidimensional cross-sectional study. Am J Gastroenterol.

[31] Pedrelli P, Feldman GC, Vorono S, Fava M, Petersen T (2008). Dysfunctional attitudes and perceived stress predict depressive symptoms severity following antidepressant treatment in patients with chronic depression. Psychiatry Res.

[32] Remor E, Penedo F, Shen B, Schneiderman N (2007). Perceived stress is associated with CD4+ cell decline in men and women living with HIV/AIDS in Spain. AIDS Care.

[33] Cohen S, Tyrrell DA, Smith AP (1993). Negative life events, perceived stress, negative affect, and susceptibility to the common cold. J Pers Soc Psychol.

[34] Cohen S, Williamson G, Spacapan S, Oskamp S (1988). Perceived stress in a probability sample of the United States. The social psychology of health: Claremont symposium on applied social psychology.

[35] Bergdahl M, Bergdahl J (2002). Perceived taste disturbance in adults: prevalence and association with oral and psychological factors and medication. Clin Oral Investig.

[36] Bergdahl J, Bergdahl M (2002). Perceived stress in adults: prevalence and association of depression, anxiety and medication in a Swedish population. Stress Health.

[37] Rönnlund M, Vestergren P, Stenling A, Nilsson LG, Bergdahl M, Bergdahl J (2015). Dimensionality of stress experiences: factorial structure of the perceived stress questionnaire (PSQ) in a population-based Swedish sample. Scand J Psychol.

[38] Karatza E, Kourou D, Galanakis M, Varvogli L, Darviri C (2014). Validation of the Greek version of perceived stress questionnaire: psychometric properties and factor structure in a population-based survey. Psychology.

[39] Fliege H, Rose M, Arck P, Levenstein S, Klapp BF (2001). Validierung des “perceived stress questionnaire”(PSQ) an einer deutschen Stichprobe. [validation of the “perceived stress questionnaire”(PSQ) in a German sample.]. Diagnostica.

[40] Sanz-Carrillo C, Garcıa-Campayo J, Rubio A, Santed M, Montoro M (2002). Validation of the Spanish version of the perceived stress questionnaire. J Psychosom Res.

[41] Wachirawat W, Hanucharurnkul S, Suriyawongpaisal P, Boonyapisit S, Levenstein S, Jearanaisilavong J (2003). Stress, but not Helicobacter pylori, is associated with peptic ulcer disease in a Thai population. J Med Assoc Thailand.

[42] Consoli S, Taine P, Szabason F, Lacour C, Metra P (1997). Development and validation of a perceived stress questionnaire recommended as a follow-up indicator in occupational medicine. L'Encephale.

[43] Saif GAB, Alotaibi HM, Alzolibani AA, Almodihesh NA, Albraidi HF, Alotaibi NM (2018). Association of psychological stress with skin symptoms among medical students. Saudi Med J.

[44] Luo Y, Gong B, Meng R, Cao X, Tang S, Fang H (2018). Validation and application of the Chinese version of the perceived stress questionnaire (C-PSQ) in nursing students. PeerJ.

[45] Lavrakas PJ. Encyclopedia of survey research methods. Sage Publications; 2008.

[46] Sidani S, Guruge S, Miranda J, Ford-Gilboe M, Varcoe C (2010). Cultural adaptation and translation of measures: an integrated method. Res Nurs Health.

[47] Sousa VD, Rojjanasrirat W (2011). Translation, adaptation and validation of instruments or scales for use in cross-cultural health care research: a clear and user-friendly guideline. J Eval Clin Pract.

[48] Beaton DE, Bombardier C, Guillemin F, Ferraz MB (2000). Guidelines for the process of cross-cultural adaptation of self-report measures. Spine.

[49] Kopp MS, Thege BK, Balog P, Stauder A, Salavecz G, Rózsa S (2010). Measures of stress in epidemiological research. J Psychosom Res.

[50] Lee E-H (2012). Review of the psychometric evidence of the perceived stress scale. Asian Nurs Res.

[51] Wang Z, Chen J, Boyd JE, Zhang H, Jia X, Qiu J (2011). Psychometric properties of the Chinese version of the perceived stress scale in policewomen. PLoS One.

[52] Ware JE, Kosinski M, Dewey JE, Gandek B. How to score and interpret single-item health status measures : a manual for users of the of the SF-8 health survey : (with a supplement on the SF-6 health survey). Lincoln, RI; Boston, MA: QualityMetric Inc.; Health Assessment Lab; 2001.

[53] Bullinger M, Alonso J, Apolone G, Leplège A, Sullivan M, Wood-Dauphinee S (1998). Translating health status questionnaires and evaluating their quality: the IQOLA project approach. J Clin Epidemiol.

[54] Wang S, Luan R, Lei Y, Kuang C, He C, Chen Y (2007). Development and evaluation of Chinese version of short form 8. Modern Prev Med.

[55] Lang L, Zhang L, Zhang P, Li Q, Bian J, Guo Y (2018). Evaluating the reliability and validity of SF-8 with a large representative sample of urban Chinese. Health Qual Life Outcomes.

[56] Goldberg D, Bridges K, Duncan-Jones P, Grayson D (1988). Detecting anxiety and depression in general medical settings. BMJ.

[57] Vergara-Romero M, Morales-Asencio JM, Morales-Fernández A, Canca-Sanchez JC, Rivas-Ruiz F, Reinaldo-Lapuerta JA (2017). Validation of the Spanish version of the Amsterdam preoperative anxiety and information scale (APAIS). Health Qual Life Outcomes.

[58] Pontin E, Schwannauer M, Tai S, Kinderman P (2013). A UK validation of a general measure of subjective well-being: the modified BBC subjective well-being scale (BBC-SWB). Health Qual Life Outcomes.

[59] Smith N. Goldberg Anxiety and Depression Inventory. Brisbane: Australian Longitudinal Study on Women's Health (ALSWH). http://www.alswh.org.au/images/content/pdf/InfoData/Data_Dictionary_Supplement/DDSSection2GADS.pdf. Accessed 16 Oct 2018..

[60] Luo Y, Meng R, Li J, Liu B, Cao X, Ge W (2019). Self-compassion may reduce anxiety and depression in nursing students: a pathway through perceived stress. Public Health.

[61] Edelen MO, Reeve BB (2007). Applying item response theory (IRT) modeling to questionnaire development, evaluation, and refinement. Qual Life Res.

[62] Wright BD, Linacre JM, Gustafsson JE, Martin-Löf P (1994). Reasonable mean-square fit values. Rasch Meas Trans.

[63] Linacre J A User’s Guide to WINSTEPS MINISTEP Rasch-Model Computer Programs (Program Manual 4.4.6). Retrieved on Oct 18, 2019 from https://wwwwinstepscom/tutorialshtm.

[64] Christensen KB, Makransky G, Horton M (2017). Critical values for Yen’s Q3: identification of local dependence in the Rasch model using residual correlations. Appl Psychol Meas.

[65] Chen W-H, Revicki D, Michalos AC (2014). Differential item functioning (DIF). Encyclopedia of quality of life and well-being research.

[66] Ebel RL, Frisbie DA. Essentials of educational measurement. 5th ed. Prentice-Hall, Inc.; 1991.

[67] Keszei AP, Novak M, Streiner DL (2010). Introduction to health measurement scales. J Psychosom Res.

[68] Streiner DL, Norman GR (2006). “Precision” and “accuracy”: two terms that are neither. J Clin Epidemiol.

[69] Hair JF, Black WC, Babin BJ, Anderson RE (2014). Multivariate data analysis: Pearson new international edition.

[70] Flora DB, Curran PJ (2004). An empirical evaluation of alternative methods of estimation for confirmatory factor analysis with ordinal data. Psychol Methods.

[71] Muthén LK, Muthén BO. Mplus user’s guide. Seventh ed. Los Angeles, CA: Muthén & Muthén; 1998-2015.

[72] DeVon HA, Block ME, Moyle-Wright P, Ernst DM, Hayden SJ, Lazzara DJ (2007). A psychometric toolbox for testing validity and reliability. J Nurs Scholarsh.

[73] Kline RB (2016). Principles and practice of structural equation modeling.

[74] McDonald RP, Ho M-HR (2002). Principles and practice in reporting structural equation analyses. Psychol Methods.

[75] Hu L, Bentler PM (1999). Cutoff criteria for fit indexes in covariance structure analysis: conventional criteria versus new alternatives. Struct Equ Model Multidiscip J.

[76] DiStefano C, Liu J, Jiang N, Shi D (2018). Examination of the weighted root mean square residual: evidence for trustworthiness?. Struct Equ Model Multidiscip J.

[77] Cheung GW, Rensvold RB (2002). Evaluating goodness-of-fit indexes for testing measurement invariance. Struct Equ Model.

[78] Chen FF (2007). Sensitivity of goodness of fit indexes to lack of measurement invariance. Struct Equ Model.

[79] Meade AW, Johnson EC, Braddy PW (2008). Power and sensitivity of alternative fit indices in tests of measurement invariance. J Appl Psychol.

[80] Brannick MT (1995). Critical comments on applying covariance structure modeling. J Organ Behav.

[81] Kelloway EK (1995). Structural equation modelling in perspective. J Organ Behav.

[82] Sijtsma K, Emons WH (2011). Advice on total-score reliability issues in psychosomatic measurement. J Psychosom Res.

[83] Şimşek GG, Noyan F (2013). McDonald's ωt, Cronbach's α, and generalized θ for composite reliability of common factors structures. Commun Stat Simul Comput.

[84] Koo TK, Li MY (2016). A guideline of selecting and reporting intraclass correlation coefficients for reliability research. J Chiropr Med.

[85] McGraw KO, Wong SP (1996). Forming inferences about some intraclass correlation coefficients. Psychol Methods.

[86] Weir JP (2005). Quantifying test-retest reliability using the intraclass correlation coefficient and the SEM. J Strength Cond Res.

[87] Terwee CB, Bot SD, de Boer MR, van der Windt DA, Knol DL, Dekker J (2007). Quality criteria were proposed for measurement properties of health status questionnaires. J Clin Epidemiol.

[88] Guttman L (1945). A basis for analyzing test-retest reliability. Psychometrika.

[89] Ten Berge JM, Zegers FE (1978). A series of lower bounds to the reliability of a test. Psychometrika.

[90] Gu H, Wen Z, Fan X (2017). Structural validity of the Machiavellian personality scale: a bifactor exploratory structural equation modeling approach. Personal Individ Differ.

[91] Allen M The SAGE encyclopedia of communication research methods. Thousand Oaks, California: SAGE; 2017.

[92] Ferketich S (1991). Focus on psychometrics. Aspects of item analysis. Res Nurs Health.

[93] Cohen RJ, Swerdlik ME, Phillips SM (2009). Psychological testing and assessment: an introduction to tests and measurement.

[94] Association WM (2013). World medical association declaration of Helsinki: ethical principles for medical research involving human subjects. JAMA.

[95] MacCallum RC, Widaman KF, Zhang S, Hong S (1999). Sample size in factor analysis. Psychol Methods.

[96] Comrey AL, Lee HB (2013). A first course in factor analysis.

[97] Tabachnick BG, Fidell LS (2013). Using multivariate statistics.

[98] Kaiser HF, Rice J (1974). Little jiffy, mark IV. Educ Psychol Meas.

[99] Bond TG, Fox CM (2015). Applying the Rasch model: fundamental measurement in the human sciences.

[100] Hagquist C, Bruce M, Gustavsson JP (2009). Using the Rasch model in nursing research: an introduction and illustrative example. Int J Nurs Stud.

[101] Yu C-Y (2002). Evaluating cutoff criteria of model fit indices for latent variable models with binary and continuous outcomes.

[102] Cho E, Kim S (2015). Cronbach’s coefficient alpha: well known but poorly understood. Organ Res Methods.

[103] Davidov E, Meuleman B, Cieciuch J, Schmidt P, Billiet J (2014). Measurement equivalence in cross-national research. Annu Rev Sociol.

