# Supplementary material for: The Chinese version of the Perceived Stress Questionnaire: development and validation amongst medical students and workers
Source: Health Qual Life Outcomes. 2020 Mar 13;18:70. doi: 10.1186/s12955-020-01307-1 (PMC7071673; doi:10.1186/s12955-020-01307-1)
Supplement: Supplementary file 1 — Additional file 1 Table 1a Rasch Analysis of Item Statistics for the C-PSQ-30 (N = 2798). Table 1b Rasch Analysis of Item Statistics for the C-PSQ-17 (N = 2798). Table 1c Rasch Analysis of Item Statistics for the C-PSQ-14 (N = 2798). Table 1d Rasch Analysis of Item Statistics for the C-PSQ-13 (N = 2798). [file 12955_2020_1307_MOESM1_ESM.docx]

Table 1a Rasch Analysis of Item Statistics for the C‐PSQ-30 (N = 2,798)

| Item  No. | Mean ± SD | Item difficulty (logit) | Infit MNSQ | Outfit MNSQ | Discrimination | Item-rest correlation | If item dropped  McDonald's ω Cronbach's α | |
| --- | --- | --- | --- | --- | --- | --- | --- | --- |
| 01 | 2.094 ± 0.833 | 0.45 | 1.23 | 1.24 | 0.56 | 0.515 | 0.935 | 0.935 |
| 02 | 2.156 ± 0.811 | 0.29 | 1.13 | 1.11 | 0.56 | 0.519 | 0.935 | 0.934 |
| 03 | 2.149 ± 0.696 | 0.31 | 0.91 | 0.92 | 0.52 | 0.483 | 0.936 | 0.935 |
| 04 | 2.832 ± 0.817 | -1.29 | 1.14 | 1.16 | 0.51 | 0.466 | 0.936 | 0.935 |
| 05 | 1.608 ± 0.677 | 1.84 | 1.17 | 1.14 | 0.48 | 0.442 | 0.936 | 0.935 |
| 06 | 1.921 ± 0.743 | 0.90 | 1.02 | 1.01 | 0.57 | 0.533 | 0.935 | 0.934 |
| 07 | 2.588 ± 0.840 | -0.73 | 1.26 | 1.29 | 0.48 | 0.427 | 0.936 | 0.936 |
| 08 | 2.501 ± 0.796 | -0.53 | 0.79 | 0.78 | 0.67 | 0.650 | 0.934 | 0.933 |
| 09 | 2.496 ± 0.761 | -0.52 | 0.93 | 0.94 | 0.55 | 0.518 | 0.935 | 0.934 |
| 10 | 2.369 ± 0.814 | -0.22 | 1.10 | 1.12 | 0.55 | 0.504 | 0.936 | 0.935 |
| 11 | 2.586 ± 0.762 | -0.73 | 1.25 | 1.37 | **0.37** | 0.319 | 0.937 | 0.937 |
| 12 | 1.965 ± 0.683 | 0.79 | 0.73 | 0.72 | 0.64 | 0.616 | 0.934 | 0.933 |
| 13 | 2.370 ± 0.827 | -0.23 | 1.03 | 1.04 | 0.60 | 0.564 | 0.935 | 0.934 |
| 14 | 2.193 ± 0.682 | 0.20 | 0.74 | 0.75 | 0.59 | 0.567 | 0.935 | 0.934 |
| 15 | 2.273 ± 0.812 | 0.00 | 0.89 | 0.89 | 0.66 | 0.636 | 0.934 | 0.933 |
| 16 | 2.470 ± 0.860 | -0.46 | 1.08 | 1.10 | 0.60 | 0.567 | 0.935 | 0.934 |
| 17 | 2.512 ± 0.891 | -0.56 | 1.34 | 1.36 | 0.52 | 0.468 | 0.936 | 0.935 |
| 18 | 2.325 ± 0.743 | -0.12 | 0.64 | 0.63 | 0.71 | 0.695 | 0.933 | 0.932 |
| 19 | 2.267 ± 0.745 | 0.02 | 0.76 | 0.76 | 0.65 | 0.627 | 0.934 | 0.933 |
| 20 | 1.921 ± 0.717 | 0.90 | 0.80 | 0.80 | 0.65 | 0.623 | 0.934 | 0.933 |
| 21 | 2.184 ± 0.808 | 0.22 | 0.99 | 0.99 | 0.63 | 0.592 | 0.935 | 0.934 |
| 22 | 2.080 ± 0.803 | 0.48 | 1.05 | 1.03 | 0.60 | 0.568 | 0.935 | 0.934 |
| 23 | 2.527 ± 0.828 | -0.59 | 1.13 | 1.15 | 0.53 | 0.494 | 0.936 | 0.935 |
| 24 | 1.853 ± 0.676 | 1.09 | 0.83 | 0.84 | 0.59 | 0.560 | 0.935 | 0.934 |
| 25 | 2.857 ± 0.880 | -1.34 | 1.19 | 1.18 | 0.57 | 0.520 | 0.935 | 0.934 |
| 26 | 2.320 ± 0.802 | -0.11 | 0.75 | 0.74 | 0.72 | 0.698 | 0.933 | 0.932 |
| 27 | 2.150 ± 0.857 | 0.31 | 0.94 | 0.92 | 0.72 | 0.695 | 0.933 | 0.932 |
| 28 | 1.924 ± 0.825 | 0.90 | 0.97 | 0.94 | 0.70 | 0.685 | 0.933 | 0.932 |
| 29 | 2.662 ± 0.871 | -0.90 | 1.31 | 1.35 | 0.49 | 0.443 | 0.936 | 0.935 |
| 30 | 2.427 ± 0.778 | -0.36 | 0.82 | 0.82 | 0.64 | 0.615 | 0.934 | 0.933 |

Abbreviation: SD = standard deviation, Infit = information-weighted fit statistic, Outfit = outlier-sensitive fit statistics, MNSQ = mean square

Discrimination index of item 11 (in bold) was less than 0.40.

Table 1b Rasch Analysis of Item Statistics for the C‐PSQ-17 (N = 2,798)

| Item  No. | Item difficulty (logit) | Infit MNSQ | Outfit MNSQ | Discrimination | Item-rest correlation | If item dropped  McDonald's ω Cronbach's α | |
| --- | --- | --- | --- | --- | --- | --- | --- |
| 03 | 0.15 | 1.05 | 1.04 | 0.55 | 0.486 | 0.909 | 0.907 |
| 04 | -1.71 | 1.21 | 1.26 | 0.55 | 0.477 | 0.909 | 0.908 |
| 05 | 1.97 | 1.29 | 1.26 | 0.52 | 0.458 | 0.909 | 0.908 |
| 06 | 0.86 | 1.14 | 1.11 | 0.61 | 0.554 | 0.907 | 0.905 |
| 08 | -0.85 | 0.91 | 0.91 | 0.67 | 0.621 | 0.905 | 0.903 |
| 09 | -0.84 | 1.01 | 1.02 | 0.60 | 0.536 | 0.907 | 0.906 |
| 12 | 0.72 | 0.82 | 0.80 | 0.67 | 0.623 | 0.905 | 0.903 |
| 14 | 0.01 | 0.82 | 0.84 | 0.62 | 0.582 | 0.906 | 0.904 |
| 15 | -0.22 | 0.95 | 0.95 | 0.71 | 0.661 | 0.904 | 0.902 |
| 16 | -0.76 | 1.15 | 1.19 | 0.65 | 0.584 | 0.906 | 0.904 |
| 18 | -0.37 | 0.70 | 0.69 | 0.73 | 0.698 | 0.902 | 0.901 |
| 19 | -0.20 | 0.81 | 0.80 | 0.69 | 0.652 | 0.904 | 0.902 |
| 20 | 0.86 | 0.92 | 0.90 | 0.67 | 0.622 | 0.905 | 0.903 |
| 23 | -0.92 | 1.29 | 1.33 | 0.55 | 0.474 | 0.909 | 0.908 |
| 24 | 1.09 | 0.94 | 0.92 | 0.62 | 0.572 | 0.906 | 0.905 |
| 28 | 0.85 | 1.16 | 1.11 | 0.70 | 0.660 | 0.904 | 0.902 |
| 30 | -0.65 | 0.89 | 0.89 | 0.67 | 0.624 | 0.905 | 0.903 |

Abbreviation: SD = standard deviation, Infit = information-weighted fit statistic, Outfit = outlier-sensitive fit statistics, MNSQ = mean square

Table 1c Rasch Analysis of Item Statistics for the C‐PSQ-14 (N = 2,798)

| Item  No. | Item difficulty (logit) | Infit MNSQ | Outfit MNSQ | Discrimination | Item-rest correlation | If item dropped  McDonald's ω Cronbach's α | |
| --- | --- | --- | --- | --- | --- | --- | --- |
| 03 | 0.27 | 1.03 | 1.02 | 0.56 | 0.488 | 0.884 | 0.881 |
| 04 | -1.61 | 1.18 | 1.21 | 0.57 | 0.475 | 0.885 | 0.882 |
| 05 | 2.13 | 1.29 | 1.26 | 0.52 | 0.446 | 0.885 | 0.883 |
| 06 | 1.00 | 1.14 | 1.12 | 0.62 | 0.545 | 0.881 | 0.879 |
| 08 | -0.74 | 0.89 | 0.88 | 0.68 | 0.622 | 0.878 | 0.875 |
| 09 | -0.72 | 0.98 | 0.98 | 0.61 | 0.537 | 0.882 | 0.879 |
| 12 | 0.86 | 0.84 | 0.82 | 0.67 | 0.607 | 0.878 | 0.876 |
| 14 | 0.14 | 0.83 | 0.84 | 0.63 | 0.568 | 0.880 | 0.878 |
| 16 | -0.65 | 1.15 | 1.19 | 0.65 | 0.573 | 0.880 | 0.878 |
| 18 | -0.25 | 0.71 | 0.70 | 0.74 | 0.691 | 0.874 | 0.872 |
| 19 | -0.08 | 0.82 | 0.81 | 0.69 | 0.641 | 0.877 | 0.874 |
| 20 | 1.00 | 0.95 | 0.94 | 0.66 | 0.601 | 0.878 | 0.876 |
| 23 | -0.81 | 1.28 | 1.32 | 0.55 | 0.463 | 0.885 | 0.883 |
| 30 | -0.54 | 0.89 | 0.90 | 0.67 | 0.612 | 0.878 | 0.876 |

Abbreviation: SD = standard deviation, Infit = information-weighted fit statistic, Outfit = outlier-sensitive fit statistics, MNSQ = mean square

Table 1d Rasch Analysis of Item Statistics for the C‐PSQ-13 (N = 2,798)

| Item  No. | Item difficulty (logit) | Infit MNSQ | Outfit MNSQ | Discrimination | Item-rest correlation | If item dropped  McDonald's ω Cronbach's α | |
| --- | --- | --- | --- | --- | --- | --- | --- |
| 03 | 0.23 | 1.04 | 1.03 | 0.57 | 0.485 | 0.876 | 0.873 |
| 04 | -1.69 | 1.21 | 1.27 | 0.56 | 0.456 | 0.877 | 0.875 |
| 05 | 2.13 | 1.29 | 1.26 | 0.53 | 0.453 | 0.877 | 0.874 |
| 06 | 0.97 | 1.16 | 1.13 | 0.63 | 0.548 | 0.872 | 0.869 |
| 08 | -0.81 | 0.92 | 0.91 | 0.68 | 0.610 | 0.869 | 0.866 |
| 09 | -0.79 | 0.99 | 0.98 | 0.62 | 0.539 | 0.873 | 0.870 |
| 12 | 0.82 | 0.84 | 0.82 | 0.68 | 0.613 | 0.869 | 0.866 |
| 14 | 0.09 | 0.86 | 0.87 | 0.63 | 0.562 | 0.871 | 0.869 |
| 18 | -0.31 | 0.72 | 0.71 | 0.74 | 0.688 | 0.864 | 0.862 |
| 19 | -0.14 | 0.83 | 0.81 | 0.70 | 0.642 | 0.867 | 0.864 |
| 20 | 0.97 | 0.96 | 0.94 | 0.67 | 0.606 | 0.869 | 0.866 |
| 23 | -0.88 | 1.30 | 1.34 | 0.56 | 0.460 | 0.877 | 0.875 |
| 30 | -0.60 | 0.92 | 0.92 | 0.67 | 0.603 | 0.870 | 0.866 |

Abbreviation: SD = standard deviation, Infit = information-weighted fit statistic, Outfit = outlier-sensitive fit statistics, MNSQ = mean square
