# Supplementary material for: The Chinese version of the Perceived Stress Questionnaire: development and validation amongst medical students and workers
Source: Health Qual Life Outcomes. 2020 Mar 13;18:70. doi: 10.1186/s12955-020-01307-1 (PMC7071673; doi:10.1186/s12955-020-01307-1)
Supplement: Supplementary file 2 — Additional file 2. Table 2a Differential Item Functioning of the C-PSQ-30 across Subgroups. Table 2b Differential Item Functioning of the C-PSQ-17 across Subgroups. Table 2c Differential Item Functioning of the C-PSQ-14 across Subgroups. Table 2d Differential Item Functioning of the C-PSQ-13 across Subgroups. [file 12955_2020_1307_MOESM2_ESM.docx]

Table 2a Differential Item Functioning of the C‐PSQ-30 across Subgroups

| Item  No. | DIF contrast  logit | Mantel–Haenszel  chi-square *P* | | DIF Measure  medical students medical workers | |
| --- | --- | --- | --- | --- | --- |
| 01 | 0.00 | 0.22 | 0.640 | 0.45 | 0.45 |
| 02 | **0.84** | 100.00 | <0.001 | 0.68 | -0.16 |
| 03 | -0.17 | 1.50 | 0.221 | 0.24 | 0.40 |
| 04 | 0.34 | 38.86 | <0.001 | -1.15 | -1.48 |
| 05 | -0.15 | 1.34 | 0.247 | 1.77 | 1.92 |
| 06 | -0.29 | 19.02 | <0.001 | 0.78 | 1.07 |
| 07 | **-0.59** | 59.69 | <0.001 | -0.99 | -0.40 |
| 08 | 0.34 | 25.83 | <0.001 | -0.38 | -0.72 |
| 09 | -0.35 | 30.28 | <0.001 | -0.67 | -0.32 |
| 10 | **-0.52** | 60.93 | <0.001 | -0.45 | 0.07 |
| 11 | -0.19 | 0.12 | 0.726 | -0.81 | -0.62 |
| 12 | 0.00 | 0.00 | 0.972 | 0.79 | 0.79 |
| 13 | -0.15 | 5.85 | 0.016 | -0.29 | -0.14 |
| 14 | -0.23 | 11.82 | 0.001 | 0.10 | 0.33 |
| 15 | -0.44 | 90.96 | <0.001 | -0.19 | 0.25 |
| 16 | 0.40 | 38.55 | <0.001 | -0.28 | -0.68 |
| 17 | -0.09 | 0.40 | 0.529 | -0.60 | -0.51 |
| 18 | -0.19 | 36.23 | <0.001 | -0.20 | -0.01 |
| 19 | -0.09 | 5.50 | 0.019 | -0.02 | 0.07 |
| 20 | 0.00 | 0.00 | 0.985 | 0.90 | 0.90 |
| 21 | 0.11 | 2.50 | 0.114 | 0.27 | 0.16 |
| 22 | **-0.58** | 98.03 | <0.001 | 0.23 | 0.81 |
| 23 | -0.17 | 5.07 | 0.024 | -0.67 | -0.50 |
| 24 | 0.41 | 60.15 | <0.001 | 1.28 | 0.88 |
| 25 | -0.07 | 0.54 | 0.463 | -1.37 | -1.31 |
| 26 | **0.50** | 54.46 | <0.001 | 0.12 | -0.38 |
| 27 | **0.60** | 55.29 | <0.001 | 0.58 | -0.02 |
| 28 | 0.49 | 22.25 | <0.001 | 1.12 | 0.63 |
| 29 | 0.46 | 61.90 | <0.001 | -0.70 | -1.16 |
| 30 | -0.10 | 7.84 | 0.005 | -0.41 | -0.30 |

Abbreviation: DIF = differential item functioning

DIF across subgroups (medical students, N =1,575 and medical workers, N = 1,223)

DIF contrast logit values (in bold) for items 02, 07, 10, 22, 26 and 27 were higher than or equal to 0.50.

Table 2b Differential Item Functioning of the C‐PSQ-17 across Subgroups

| Item  No. | DIF contrast  logit | Mantel–Haenszel  chi-square *P* | | DIF Measure  medical students medical workers | |
| --- | --- | --- | --- | --- | --- |
| 03 | -0.17 | 1.33 | 0.248 | 0.07 | 0.24 |
| 04 | 0.36 | 42.50 | <0.001 | -1.56 | -1.92 |
| 05 | -0.15 | 1.45 | 0.228 | 1.91 | 2.06 |
| 06 | -0.31 | 20.40 | <0.001 | 0.72 | 1.04 |
| 08 | 0.39 | 40.52 | <0.001 | -0.67 | -1.07 |
| 09 | -0.41 | 32.33 | <0.001 | -1.01 | -0.61 |
| 12 | 0.00 | 0.04 | 0.844 | 0.72 | 0.72 |
| 14 | -0.25 | 10.96 | 0.001 | -0.10 | 0.15 |
| 15 | **-0.51** | 93.36 | <0.001 | -0.44 | 0.06 |
| 16 | 0.47 | 43.36 | <0.001 | -0.55 | -1.02 |
| 18 | -0.21 | 23.55 | <0.001 | -0.46 | -0.25 |
| 19 | -0.09 | 3.63 | 0.057 | -0.24 | -0.15 |
| 20 | 0.07 | 0.06 | 0.801 | 0.89 | 0.82 |
| 23 | -0.20 | 3.32 | 0.069 | -1.01 | -0.81 |
| 24 | **0.53** | 70.17 | <0.001 | 1.33 | 0.80 |
| 28 | **0.63** | 39.57 | <0.001 | 1.14 | 0.52 |
| 30 | -0.12 | 4.97 | 0.026 | -0.70 | -0.58 |

Abbreviation: DIF = differential item functioning

DIF across subgroups (medical students, N =1,575 and medical workers, N = 1,223)

DIF contrast logit values (in bold) for items 15, 24 and 28 were higher than or equal to 0.50.

Table 2c Differential Item Functioning of the C‐PSQ-14 across Subgroups

| Item  No. | DIF contrast  logit | Mantel–Haenszel  chi-square *P* | | DIF Measure  medical students medical workers | |
| --- | --- | --- | --- | --- | --- |
| 03 | -0.13 | 1.12 | 0.291 | 0.21 | 0.35 |
| 04 | 0.38 | 43.32 | <0.001 | -1.45 | -1.83 |
| 05 | -0.11 | 1.18 | 0.278 | 2.09 | 2.19 |
| 06 | -0.27 | 19.26 | <0.001 | 0.88 | 1.15 |
| 08 | 0.43 | 43.47 | <0.001 | -0.54 | -0.97 |
| 09 | -0.38 | 31.28 | <0.001 | -0.89 | -0.51 |
| 12 | 0.05 | 0.45 | 0.502 | 0.88 | 0.83 |
| 14 | -0.21 | 7.27 | 0.007 | 0.04 | 0.25 |
| 16 | **0.51** | 48.87 | <0.001 | -0.42 | -0.93 |
| 18 | -0.18 | 19.37 | <0.001 | -0.33 | -0.15 |
| 19 | -0.05 | 2.15 | 0.143 | -0.10 | -0.05 |
| 20 | 0.12 | 1.23 | 0.268 | 1.06 | 0.94 |
| 23 | -0.17 | 3.03 | 0.082 | -0.88 | -0.71 |
| 30 | -0.08 | 2.81 | 0.094 | -0.57 | -0.49 |

Abbreviation: DIF = differential item functioning

DIF across subgroups (medical students, N =1,575 and medical workers, N = 1,223)

DIF contrast logit values (in bold) for items 16 was higher than or equal to 0.50.

Table 2d Differential Item Functioning of the C‐PSQ-13 across Subgroups

| Item  No. | DIF contrast  logit | Mantel–Haenszel  chi-square *P* | | DIF Measure  medical students medical workers | |
| --- | --- | --- | --- | --- | --- |
| 01 | -0.09 | 0.06 | 0.803 | 0.19 | 0.28 |
| 02 | 0.42 | 51.14 | <0.001 | -1.51 | -1.94 |
| 03 | -0.07 | 0.49 | 0.485 | 2.10 | 2.17 |
| 04 | -0.24 | 15.09 | <0.001 | 0.87 | 1.11 |
| 05 | 0.48 | 51.93 | <0.001 | -0.59 | -1.07 |
| 06 | -0.34 | 26.10 | <0.001 | -0.95 | -0.60 |
| 07 | 0.09 | 1.83 | 0.177 | 0.87 | 0.78 |
| 08 | -0.17 | 3.97 | 0.046 | 0.01 | 0.18 |
| 09 | -0.14 | 12.78 | <0.001 | -0.37 | -0.23 |
| 10 | 0.00 | 0.45 | 0.504 | -0.14 | -0.14 |
| 11 | 0.17 | 2.69 | 0.101 | 1.05 | 0.89 |
| 12 | -0.13 | 1.35 | 0.246 | -0.94 | -0.80 |
| 13 | -0.02 | 1.76 | 0.185 | -0.60 | -0.58 |

Abbreviation: DIF = differential item functioning

DIF across subgroups (medical students, N =1,575 and medical workers, N = 1,223)
